# Genetic Polymorphisms in the Hypothalamic Pathway in Relation to Subsequent Weight Change – The DiOGenes Study

**DOI:** 10.1371/journal.pone.0017436

**Published:** 2011-02-24

**Authors:** Huaidong Du, Karani S. Vimaleswaran, Lars Ängquist, Rikke D. Hansen, Daphne L. van der A, Claus Holst, Anne Tjønneland, Kim Overvad, Marianne Uhre Jakobsen, Heiner Boeing, Karina Meidtner, Domenico Palli, Giovanna Masala, Nabila Bouatia-Naji, Wim H. M. Saris, Edith J. M. Feskens, Nicolas J.Wareham, Thorkild I. A. Sørensen, Ruth J. F. Loos

**Affiliations:** 1 National Institute for Public Health and the Environment (RIVM), Bilthoven, The Netherlands; 2 Department of Human Biology, Nutrition and Toxicology Research Institute of Maastricht (NUTRIM), Maastricht, The Netherlands; 3 Medical Research Council (MRC) Epidemiology Unit, Institute of Metabolic Science, Cambridge, United Kingdom; 4 Institute of Preventive Medicine, Copenhagen University Hospital, Copenhagen, Denmark; 5 Danish Cancer Society, Institute of Cancer Epidemiology, Copenhagen, Denmark; 6 Department of Cardiology, Aalborg Hospital, Aarhus University Hospital, Aalborg, Denmark; 7 Department of Clinical Epidemiology, Aarhus University Hospital, Aalborg, Denmark; 8 Department of Epidemiology, German Institute of Human Nutrition, Potsdam, Germany; 9 Molecular and Nutritional Epidemiology Unit, Cancer Research and Prevention Institute (ISPO), Florence, Italy; 10 Université Lille-Nord de France, Lille, France; 11 CNRS UMR 8199, Institut Pasteur de Lille, Lille, France; 12 Division of Human Nutrition, Wageningen University, Wageningen, The Netherlands; National Institutes of Health - National Institute of Child Health and Human Development, United States of America

## Abstract

**Background:**

Single nucleotide polymorphisms (SNPs) in genes encoding the components involved in the hypothalamic pathway may influence weight gain and dietary factors may modify their effects.

**Aim:**

We conducted a case-cohort study to investigate the associations of SNPs in candidate genes with weight change during an average of 6.8 years of follow-up and to examine the potential effect modification by glycemic index (GI) and protein intake.

**Methods and Findings:**

Participants, aged 20–60 years at baseline, came from five European countries. Cases (‘weight gainers’) were selected from the total eligible cohort (n = 50,293) as those with the greatest unexplained annual weight gain (n = 5,584). A random subcohort (n = 6,566) was drawn with the intention to obtain an equal number of cases and noncases (n = 5,507). We genotyped 134 SNPs that captured all common genetic variation across the 15 candidate genes; 123 met the quality control criteria. Each SNP was tested for association with the risk of being a ‘weight gainer’ (logistic regression models) in the case-noncase data and with weight gain (linear regression models) in the random subcohort data. After accounting for multiple testing, none of the SNPs was significantly associated with weight change. Furthermore, we observed no significant effect modification by dietary factors, except for SNP rs7180849 in the neuromedin β gene (*NMB*). Carriers of the minor allele had a more pronounced weight gain at a higher GI (*P* = 2×10^−7^).

**Conclusions:**

We found no evidence of association between SNPs in the studied hypothalamic genes with weight change. The interaction between GI and *NMB* SNP rs7180849 needs further confirmation.

## Introduction

The hypothalamic pathway plays a critical role in the development of obesity through regulating appetite and satiety, food intake, and thus energy balance [1]. The hypothalamus integrates gastrointestinal signals such as cholecystokinin (CCK) and ghrelin, adiposity signals such as leptin, and hypothalamic signals such as alpa-melanocyte-stimulating hormone (α-MSH) and its precursor pro-opiomelanocortin (POMC) and neuropeptide Y (NPY) [1], [2]. Most of the genes encoding these signals have been proposed as obesity susceptibility genes [3], [4], although findings tend to be inconsistent [3]. For instance, common polymorphisms in the melanocortin-4 receptor gene (*MC4R*) have been associated with human obesity in two candidate gene meta-analyses [5], [6]. Moreover, several common variants near hypothalamic genes such as *MC4R* and *POMC* have been identified associated with body mass index (BMI) in a recent genome-wide association study [7]. So far, most candidate genes studies have been small and often insufficiently powered to identify the small effects expected. Furthermore, most studies have focused on concurrent BMI or obesity, whereas only few have aimed at identifying associations with weight change [8].

In spite of the evidence for a substantial heritable component to obesity [9], there is no doubt that the recent obesity epidemic is due to major changes in our living environment, possibly mediated through an altered lifestyle. During the past decades, many dietary factors have been investigated for their potential impacts on weight change or obesity, such as protein intake and dietary glycemic index (GI) [10], [11]. Most, but not all, studies suggest that diets high in protein and low in GI are beneficial for obesity prevention and weight control [10], [11]. It has been suggested that these diets enhance satiety, leading to a decreased energy intake, thus prevent weight gain and promote weight loss [10], [11].

The aims of the current case-cohort study were to investigate whether single nucleotide polymorphisms (SNP) within or near genes involved in the hypothalamic pathway are associated with weight gain and to examine whether protein intake and dietary GI modify their effects. The results will be of relevance for both understanding the etiology of weight gain and for tailoring dietary prevention interventions on obesity.

## Methods

### Ethics statement

EPIC study has been approved by local review board of all participating institutions, namely the Florence Local Health Authority Ethical Committee (Italy), the Ethics Committee of the Norwich District Health Authority (UK), the Medical Ethics Committee of TNO (Netherlands Organisation for Applied Scientific Research) (the Netherlands), the Ethics Committee of the Medical Association of the State of Brandenburg (Germany), and the Danish National Committee on Biomedical Research Ethics (Denmark). Written informed consent has been obtained from all participants before joining EPIC study.

### Participants

**Figure 1:**
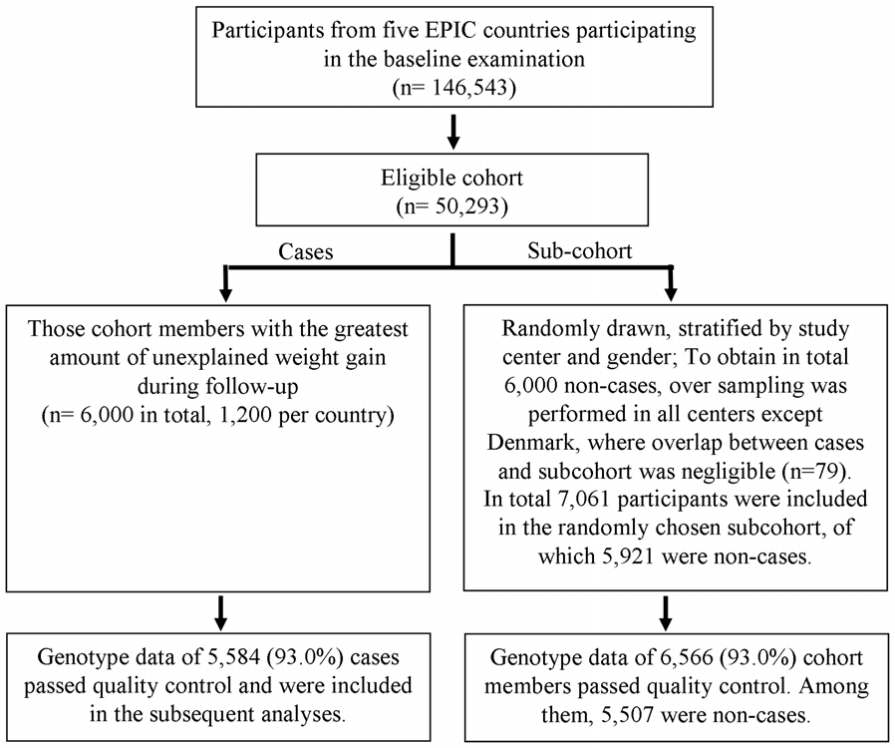
Flow diagram of participant selection.

Participants came from cohorts established in eight regions within five European countries (Italy, UK, the Netherlands, Germany, Denmark) participating in the European Prospective Investigation into Cancer and Nutrition (EPIC) study [12]. The cohorts were those in the EPIC that had a follow-up program including reassessment of anthropometry completed. Individuals were eligible if the following inclusion criteria were met: younger than 60 years of age at baseline and younger than 65 years at follow-up, blood sample available, had baseline information on diet, weight and height and follow-up information on weight, stable smoking habits, no cancer, cardiovascular diseases (CVD), and diabetes, and an annual weight change not more than 5 kg/year. Of the 146,543 participants who took part in the baseline examination during 1992–1998, a total of 50,293 men and women were eligible to participate in our study. Cases were defined as those individuals who had experienced the greatest degree of unexplained annual weight gain during follow-up (with an average duration of 6.8 years). They were identified by using the residuals from a regression model of annual weight change on baseline values of age, weight and height, smoking status (current/former/never smokers), and follow-up time. Regression models were run separately for each country and stratified by gender. For each of the five countries, except Italy, we randomly selected 600 male and 600 female cases. As the Italian cohort consisted of a general population-based sample and of a women-only sample (population-based breast cancer screening program), men were underrepresented (27%). Consistent with the sex-ratio in the Italian cohort, we selected 300 male and 900 female cases. The subcohort sample comprised a random sample of the total eligible cohort and was drawn in such a way that the total number of noncases equaled the number of cases (n = 1,200 in each country). This resulted in some cases also selected for the random subcohort. Therefore, oversampling of the random subcohort was performed, except in Denmark where overlap between cases and subcohort was negligible (n = 79). In total, 11,921 participants were included in the present genetic association study: 6,000 cases and a subcohort of 7,061 individuals, of which 5,921 were noncases. The flow chart of participant selection is shown in **Figure 1**. The demographic, anthropometric and dietary characteristics of cases, noncases and random subcohort are presented in **Table 1**.

**Table 1:** Characteristics of participants of cases, noncases and subcohort.

	Cases	Noncases	*P* values^1^	Subcohort
	(n = 5,584)	(n = 5,507)		(n = 6,566)
Age, *yrs*	47.6±7.5	48.0±7.3	0.003	47.9±7.3
Gender, *%men*	45	45	matched	46
Overweight, *%*	43	39	<0.0001	39
Obesity, *%*	17	9	<0.0001	10
Baseline weight, *kg*	76.3±14.3	72.6±13.4	<0.0001	73.2±13.6
Baseline BMI, *kg/m^2^*	26.4±4.2	25.2±3.6	<0.0001	25.4±3.7
Annual weight change, *g/yr*	1,428±684	30±622	<0.0001	245±801
BMI at follow-up, *kg/m^2^*	29.4±4.4	25.3±3.5	<0.0001	25.9±3.9
Follow-up time, *yrs*	6.8±2.5	6.8±2.5	0.08	6.9±2.5
Glycemic index (GI)	56.6±4.3	56.5±4.1	0.4	56.5±4.1
Protein intake, *g*	89.9±29.4	89.2±27.1	0.2	89.6±28.2

Values presented are mean ± standard deviation or percentage (%) as indicated.

1 *P* values for the difference between cases and noncases, tested by student t-test (for continuous variables) or Cochran-Armitage trend test (categorical variables).

### Measurements of diet, anthropometrics and smoking status

Validated country-specific food frequency questionnaires (FFQs) were used to collect dietary information at baseline [12]. GI and protein intake were assessed using the methods described earlier [13], [14].

Details of the anthropometric measurements have been described previously [13], [14]. In brief, at baseline all participants were measured for weight and height using standard study protocols [15]. At follow-up, participants in the UK and one center in the Netherlands (Doetinchem) were measured again by trained technicians, while all other participants measured their weight at home according to the guidance provided. Therefore participants from Doetinchem were analyzed separately from other Dutch participants. As such, we analyzed the data by six study centers in five countries.

Information on smoking status (never, former, or current smoker) was collected via self-administered questionnaires at baseline and at follow-up. Only those who had not changed their smoking habits during follow-up were included in the analyses.

### Selection of candidate genes and tagSNPs

We selected 15 candidate genes based on the available evidence from literature on their potential roles in body weight regulation through mechanisms presumed to be implicated in the hypothalamic pathway, either directly or via the interactions with the dietary GI and protein intake [3]. These genes encode gastrointestinal signals, adiposity signals and hypothalamic signals of the hypothalamic pathway, including cholecystokinin (CCK), CCK A receptor (CCKAR), ghrelin (GHRL), glucagon-like peptide-1 (GLP-1), peptide YY3-36 (PYY) [16], leptin (LEP), leptin receptor (LEPR)[17], interleukin-6 (IL-6)[18], neuropeptide Y (NPY)[19], proopiomelanocortin (POMC)[20], melanocortin 4 receptor (MC4R)[5], nucleobindin 2 (NUCB2)[21], mammalian target of rapamycin (mTOR) [22], neuromedin β (NMB)[23], and 5-hydroxytryptamine (serotonin) receptor 1A (5-HT_1A_)[24]. A figure summarizing the interrelationship between many of the hypothalamic hormones in relation to their role in the regulation of food intake can be found in a recent review [1].

We used the International HapMap data for European ancestry (CEU) (release 20, NCBI Build 35) to select SNPs such that full coverage of the common genetic variation in the candidate genes (+/−5 kb) was ensured.

The Haploview software V3.3 was used to assess the linkage disequilibrium (LD) structure between SNPs [25]. Tagger software was used to select tagSNPs with the ‘pairwise tagging only’ option and an r^2^ threshold of 0.8. In total, 134 SNPs were selected using the pre-requisite criteria (minor allele frequency (MAF) ≥5% and Hardy-Weinberg Equilibrium (HWE) *P*>0.01). A complete list of the selected SNPs, including the candidate genes, is shown in a **Table S1**.

### DNA extraction and genotyping

Genomic DNA was extracted from the buffy coats with a salting out method [26], except for participants from the UK, for whom whole-genome amplified DNA was used. Genomic and amplified DNA samples were quality-checked, quantified and normalized to approximately 100 ng/ml and 2.0 µg before genotyping. High throughput SNP genotyping was carried out using the Illumina® Beadstation Genotyping System at IntegraGen, France.

Genotyping was considered successful if the following quality control criteria were met within each country: sample call rate ≥95%, SNP call rate ≥95%, and duplicate discordance rate ≤3%.

We subjected all SNPs to country-specific HWE genotype distribution-tests. Significant deviations from equilibrium were defined as *P*≤0.001 and then lead to exclusion of that particular SNP-country genotype data. In total, 123 SNPs passed the tests for at least one country and were successfully genotyped for 11,091 participants. The case group included 5,584 participants and the subcohort included 6,566 participants of whom 5,507 were noncases.

### Statistical methods

Power calculations were performed using QUANTO software, Version 1.2.4 (May 2009) [27]. The minimum detectable effects, at 80% power, ranged from OR (odds ratio) 1.44 for a SNP with a MAF of 5% to OR 1.08 for a SNP with a MAF 50% in the case-noncase analyses and from regression coefficient (β) 88 g/year to β 40 g/year in random subcohort analyses.

Each SNP was coded 0, 1 and 2 according to the number of minor alleles an individual carries.

First, the association between each SNP and BMI at baseline was tested using linear regression, assuming an additive effect of the minor allele. Analyses were adjusted for age, gender, height and smoking status.

Subsequently, we tested for association with weight gain over time. The association of each SNP with the risk of being a ‘weight gainer’ (case-noncase analysis) was examined using a logistic regression assuming an additive effect of the minor allele. The association between each SNP and annual weight change (g/year) (random subcohort analysis) was tested using linear regression and assuming an additive effect of the minor allele.

Case-noncase analyses of main effects were not adjusted, whereas random subcohort analyses were adjusted for variables that had been included in the case-status defining model (i.e. baseline values of age, weight and height, gender, smoking status, and follow-up time) to reduce the residual variation.

SNP-dietary factor interaction analyses were performed by including the corresponding interaction term as well as the complementary dietary main effect term in the model.

All association analyses were first conducted for each study center separately and then summary statistics were meta-analyzed. We used random effects to account for the possible heterogeneity across study centers. Heterogeneity across study centers was tested using the Cochran Q-test [28].

Multiple comparisons were corrected for using the Bonferroni method. Number of tests corrected for was chosen as the number of SNPs studied (n = 123) and *P*<4×10^−4^ (0.05/123) was considered significant.

All association analyses were conducted using STATA 9.2 for Windows (StataCorp LP, Texas, USA). The descriptive analyses were performed with SAS 9.1 for Windows (SAS Institute, Cary, NC).

## Results

None of the associations between the 123 SNPs and baseline BMI in the subcohort was significant after accounting for multiple testing, yet three associations reached nominal significance (*P*<0.05) (*LEPR* SNP rs3790426 β = 0.18 kg/m^2^ per minor allele, *P* = 0.01; *LEP* SNP rs2278815 β = −0.14 kg/m^2^ per minor allele, *P* = 0.03; *NUCB2* SNP rs10832763 β = 0.16 kg/m^2^ per minor allele, *P* = 0.03) (**Table S2**).

In case-noncase analyses, none of the associations or interactions under investigation were considered significant after the correction for multiple testing. However, two SNPs showed a nominally significant association with the risk of being a ‘weight gainer’ (*PYY* SNP rs1859223, OR = 0.91 per minor allele, *P* = 0.01; *CCK* SNP rs11571842, OR = 1.06 per minor allele, *P* = 0.03) (**Table S3**). The interactions between two SNPs with protein intake (*IL-6* SNP rs12700386 and *LEPR* SNP rs1887285) and between nine other SNPs and GI (*CCK* SNP rs9311317, *LEPR* SNP rs1137101, *POMC* SNP rs6713532, two *NMB* SNPs, rs2292462 and rs7180849, and four *NPY* SNPs, rs16135, rs16472, rs5574, and rs9785023) reached nominal significance level (*P*<0.05) (**Table S4**).

Again, in the random subcohort analyses, none of the SNP-weight change associations was significant after multiple testing correction, in spite of the three nominally significant associations of *LEPR* SNP rs10493380 (β = −30 g/year per minor allele, *P* = 0.04), *PYY* SNP rs1058046 (β = − 28 g/year per minor allele, *P* = 0.02), and *GHRL* SNP rs26802 (β = 25 g/year per minor allele, *P* = 0.04) with weight change (**Table S5**).

**Figure 2:**
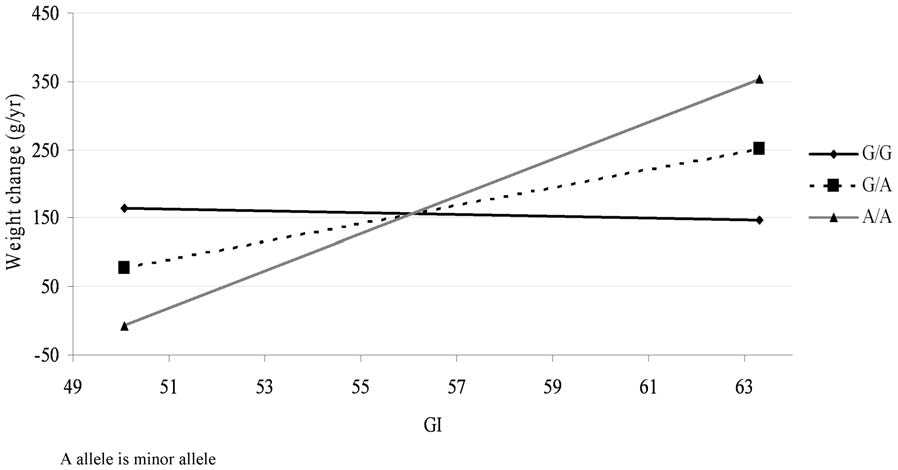
The regression of weight change on glycemic index (GI) by genotypes of SNP rs7180849 near neuromedin β gene gene (*NMB*) (n = 5,117 - distributed over genotypes as GG = 3,549, AG = 1,431, and AA = 137) .

None of the SNP-GI and SNP-protein interaction were found to be significant after multiple testing correction, apart from the interaction between the *NMB* rs7180849 SNP and GI remained significant (β = 25 g/year per allele per unit increase in GI, *P* = 2×10^−7^). More specifically, the minor allele of the *NMB* rs7180849 SNP was associated with a 25 g/y greater increase in weight for every unit increase in GI. As shown in **Figure 2**, from the lowest GI quintile (mean GI ≈50) to the highest GI quintile (mean GI ≈63), the annual weight change of the participants with the AA genotype increased from −195 g/year to 526 g/year, while the weight change among those with GA genotype was much smaller (from −20 g/year to 344 g/year) and the weight change of those with the GG genotype hardly differed (from 154 g/year to 161 g/year). In addition, we also observed two nominally significant (*P*<0.05) interactions for two SNPs with protein intake (*LEP* SNP rs7788818 and *NMB* SNP rs7180849) and for eight SNPs with GI (*GHRL* SNP rs35683, *NUCB2* SNP rs214075, *POMC* SNP rs67565427, two *NMB* SNPs, rs2292462 and rs7180849, and three *LEPR* SNPs, rs1137101, rs2025805 and rs9436740) (**Table 2** **& Table S6**).

**Table 2:** Interactions of SNPs with dietary glycemic index (GI) and protein intake on weight gain in random subcohort analyses (n = 6,566).

Gene	Number of SNPs	1 minor allele×1 unit GI				1 minor allele×1 gram protein			
		β (kg/m^2^) 1		*P* value^2^		β (kg/m^2^) 1		*P* value^2^	
		Lowest	Highest	Lowest	Highest	Lowest	Highest	Lowest	Highest
*CCK*	8	−6	4	0.2	0.9	−8	10	0.2	1.0
*CCKAR*	5	−2	10	0.3	0.6	−8	2	0.2	0.8
*GHRL*	12	−8	8	0.03	1.0	−9	10	0.09	1.0
*GLP-1*	2	−8	4	0.2	0.4	−3	−2	0.7	0.8
*5-HT_1A_*	1	−4	−4	0.3	0.3	−5	−5	0.3	0.3
*IL-6*	7	−12	7	0.05	0.8	−5	12	0.1	0.9
*LEP*	6	−7	5	0.09	0.9	−30	11	0.008	0.8
*LEPR*	35	−17	11	0.02	1.0	−11	8	0.2	0.9
*MC4R*	3	0.1	7	0.2	1.0	−2	6	0.4	0.9
*mTOR*	4	−6	14	0.2	0.9	−13	7	0.3	0.8
*NMB*	4	−6	25	2×10^−7^	0.9	−20	12	0.02	0.9
*NPY*	8	−5	7	0.1	0.8	−4	4	0.6	1.0
*NUCB2*	12	−6	9	0.006	0.9	−8	12	0.06	1.0
*POMC*	9	−21	12	0.04	0.9	−9	11	0.07	1.0
*PYY*	7	−7	5	0.3	1.0	−8	0.5	0.09	1.0

SNP: single nucleotide polymorphism.

1 Values presented are the overall meta-analyzed regression coefficients. The lowest and highest values are the minimum and maximum β among the SNPs in that specific gene.

2 *P* values of the meta-analyzed βs. The lowest and highest values are the minimum and maximum *P* value among the SNPs in that specific gene.

No significant heterogeneity was observed for any of the above mentioned associations (*P*>0.2 for all).

## Discussion

In this large case-cohort study among European adults, we studied the associations of 123 SNPs in 15 candidate genes involved in the hypothalamic pathway with weight change. Despite the fact that our study has sufficient power to detect small effects, we did not observe significant associations between any of the SNPs and weight change or risk of being a ‘weight gainer’. Furthermore, dietary protein intake and GI did not modify the association between SNPs and weight change, except for the *NMB* rs7180849 that seemed to increase weight gain in those on a high GI diet.

The selection of genes for our study was based on a hypothesis-driven candidate gene approach. We selected only those genes that have a well-defined role in regulating the ‘hypothalamic pathway’, variants in which are associated with obesity-related traits. Studies in humans and animals have shown that administration of CCK, PYY, GLP-1, and NMB could decrease food intake but administration of ghrelin and NPY leads to obesity [29], [30], [31], [32]. Variants in or near *LEP*, *POMC*, *MC4R*, and *IL-6* genes confer a significant risk to human obesity [33], [34], [35]. mTOR is an important mediator between the adiposity signal, leptin, and hypothalamic signals; the inhibition of mTOR signalling blunts leptin's anorectic effect [2], [22], [36]. Serotonin systems (*5-HT_1A_*) could upregulate the expression of *NUCB2*, a hypothalamic peptide with anorexigenic effect [21], [37], and induce anorexia [38].

The critical role of food intake regulation and the hypothalamus pathway in weight change and obesity has long been recognized [1], [2]. A number of studies observed associations between genetic variations in this pathway and obesity-related phenotypes [3]. There is also evidence suggesting that dietary GI and protein intake affect food intake and weight change through satiety regulation [10], [39], [40], [41]. Several studies have shown that these dietary factors could influence the hypothalamic pathway signaling. High protein diet has been found to decrease postprandial ghrelin concentrations and increase GLP-1 and PYY release and also found to reduce gastric emptying through increased secretion of CCK and GLP-1 [42], [43]. In a recent randomized controlled trial, low GI diet has been associated with a lower day-long concentration of glucose and insulin, and a higher level of CCK [44]. However, we are not aware of any study which has examined whether associations between SNPs of hypothalamic genes and weight gain are modified by these dietary factors.

In the present study, we did not observe significant main effects of any of the SNPs on either weight change or baseline BMI. Furthermore, no significant effect modification by dietary protein was observed either. The confidence intervals of the observed estimates were narrow, reflecting a sound reliability of our findings (online supplementary tables). In the random subcohort analyses, we observed a significant interaction between GI and *NMB* SNP rs7180849; i.e. each additional minor allele of rs7180849 was associated with increased weight gain in those on a high GI diet. Although consistent with our observations for weight gain as a continuous trait, the interaction observed in the case-noncase analyses was only borderline significant after correcting for multiple testing (*P* = 0.002). We speculate that the apparent weaker interaction observed in the case-noncase analysis compared to the cohort analysis could be ascribed to the difference in study design. More specifically, despite a larger sample size, the case-noncase design does not necessarily provide more statistical power. Furthermore, the outcomes examined may represent different phenotypes; i.e. the case-noncase analysis examines association with weight gainers at the extremes of the distribution, while the cohort analysis examine weight change in the general population. In additional analyses, we also found that rs7180849 significantly interacted with GI on waist circumference change (*P* = 1.5×10^−5^ in random subcohort analyses).

**Figure 3:**
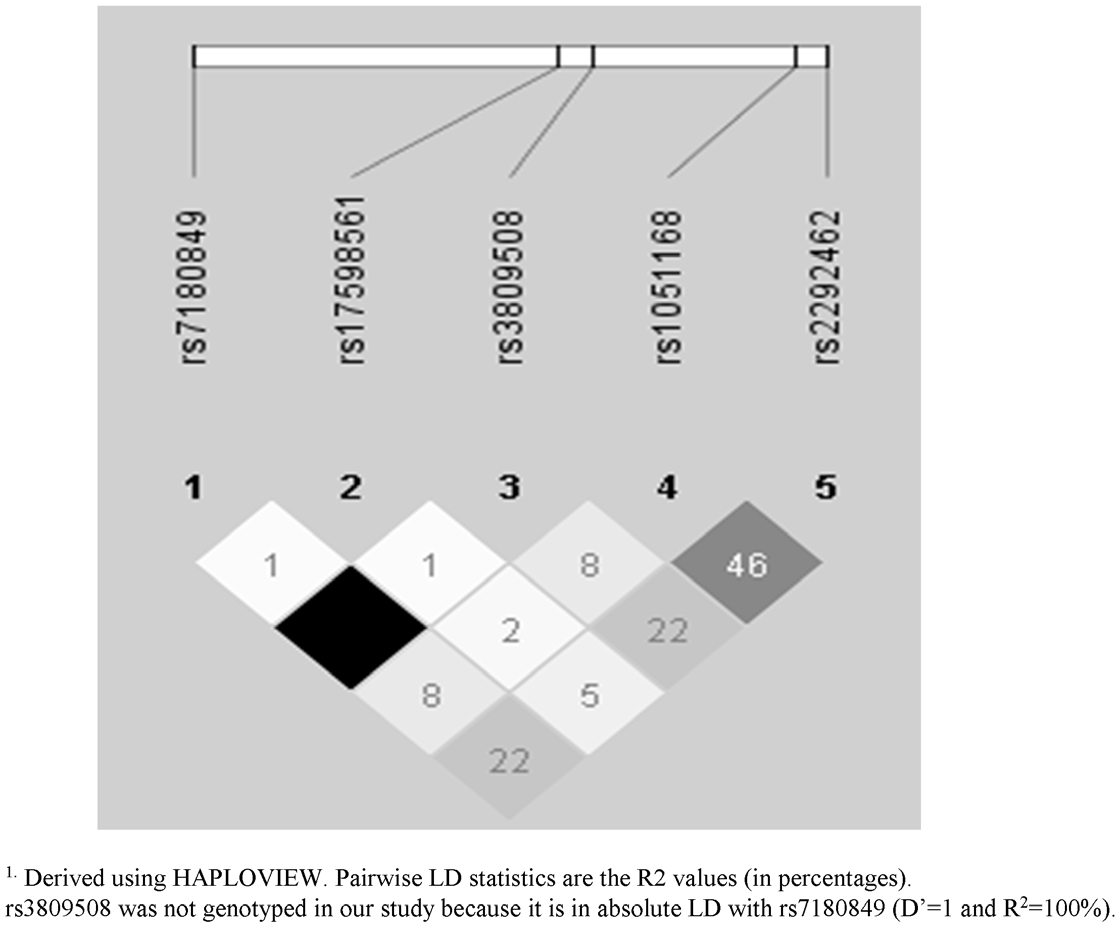
Linkage disequilibrium (LD) plot of the neuromedin β gene (*NMB*) ^1^.

NMB is a decapeptide that belongs to the bombesin-like peptide family and is widely distributed in the human central nervous system, pancreas, adrenals, gastrointestinal tract and adipose tissue [23]. Evidence shows that NMB, when released in the gastrointestinal tract in response to food intake, represents a mediator between the gut and the brain and serves as a satiation signal to terminate meals [23]. NMB is also expressed in adipose tissue and can act as an adiposity signal reflecting nutritional status and regulating food intake over a longer term [23], [45]. Intravenous infusion of NMB has been shown to reduce meal size and meal duration in rats [32]. The rs7180849 is located at ∼2.7 kb upstream of the *NMB* (**Figure 3**) and is in complete LD (r^2^ = 1.0, D′ = 1.0 in CEU Hapmap) with SNP rs3809508 which is positioned in the intron region of the *NMB*. In a recent study among 1,144 European adolescents, the TT genotype (homozygous minor allele carriers) of the rs3809508 was associated with a higher risk of obesity and this association was stronger among those adolescents whose mothers had the lowest education level [46]. Given the close association between low education and unhealthy eating habits (i.e. a high GI diet), it might be possible that the influence of SNP rs3809508 on obesity risk was, at least partly, modified by dietary GI. In the Quebec Family Study, a missense mutation p.P73T in the *NMB* has been associated with eating behaviors associated with obesity and the amount of body fat gain during a 6-year follow-up [47]. The silent polymorphism g.G401A in the *NMB* has been significantly associated with body weight in children and adolescents [48]. However, there is no identified function of either rs7180849 or rs3809508 on *NMB* expression or function so far.

The main strengths of the current study include its large scale, the multi-center and prospective properties and the full capture of common genetic variation in the candidate genes. A potential weakness might be the self-reported information on body weight at follow-up in four out of six study centers [13]. However, using this data, it has been observed in our previous studies that correction for underreporting using the available methods did not change the main findings [13].

To conclude, among this European population from five countries, none of the 123 SNPs in or near 15 candidate genes involved in the hypothalamic regulation pathway of food intake had a significant main effect on the risk of weight gain. With the exception of *NMB* SNP, rs7180849, showing a significant interaction with dietary GI, none of the SNPs showed interactions with the GI or protein content of the diet in their associations with weight change and the risk of being a ‘weight gainer’. This finding suggests that individuals carrying the *NMB* minor allele (G allele) are more vulnerable to the deleterious effects of a high GI diet in terms of weight gain. Thus, lowering dietary GI seems more important for the minor-allele carriers in weight regulation. However, further studies are needed to verify this finding.

## Supporting Information

Table S1 **List of the genes (n = 15) and single nucleotide polymorphisms (SNPs) (n = 134) selected for genotyping.**
(DOC)

Table S2 **Cross-sectional associations of 123 single nucleotide polymorphisms (SNPs) from the hypothalamic pathway with baseline body mass index (BMI, kg/m^2^).**
(DOC)

Table S3 **Association of 123 single nucleotide polymorphisms (SNPs) from the hypothalamic pathway with the risk of being a ‘weight gainer’ in case - noncase analysis (n = 11,091).**
(DOC)

Table S4 **Interaction of 123 single nucleotide polymorphisms (SNPs) from the hypothalamic pathway with glycemic index (GI) and protein intake on the risk of being a ‘weight gainer’ in case - noncase analysis (n = 11,091).**
(DOC)

Table S5 **Association of 123 single nucleotide polymorphisms (SNPs) from the hypothalamic pathway with weight gain (g/year) in random subcohort analysis (n = 6,566).**
(DOC)

Table S6 **Interactions of 123 single nucleotide polymorphisms (SNPs) from the hypothalamic pathway with glycemic index (GI) and protein intake on weight gain (g/year) in random subcohort analysis (n = 6,566).**
(DOC)
