# Supplementary material for: Genetic Polymorphisms in the Hypothalamic Pathway in Relation to Subsequent Weight Change – The DiOGenes Study
Source: PLoS One. 2011 Feb 24;6(2):e17436. doi: 10.1371/journal.pone.0017436 (PMC3044761; doi:10.1371/journal.pone.0017436)
Supplement: Table S1 — List of the genes (n = 15) and single nucleotide polymorphisms (SNPs) (n = 134) selected for genotyping. (DOC) [file pone.0017436.s001.doc]

**Table S1. List of the genes (n = 15) and single nucleotide polymorphisms (SNPs)** (n = 134) selected for genotyping.

| **Gene (chromosome)** | **SNPs** | **N^1^** | **Position** | **Major allele / Minor allele** | **MAF** |
| --- | --- | --- | --- | --- | --- |
| *CCK* (chr 3) | rs9311317 | 6 | 42270809 | A / G | 0.25 |
|  | rs10460960 | 6 | 42283738 | A / G | 0.11 |
|  | rs10865918 | 6 | 42278314 | A / C | 0.38 |
|  | rs11571842 | 6 | 42281449 | G / A | 0.49 |
|  | rs11129949 | 6 | 42277811 | A / C | 0.12 |
|  | rs747455 | 6 | 42280967 | G / A | 0.24 |
|  | rs8192472 | 6 | 42274873 | G / A | 0.38 |
|  | rs7628795 | 6 | 42270197 | G / A | 0.41 |
|  | rs11129947 | 0 |  |  | Failed |
| *CCKAR* (chr 4) | rs2000978 | 6 | 26097086 | A / G | 0.17 |
|  | rs7665027 | 6 | 26096006 | A / G | 0.15 |
|  | rs1573596 | 6 | 26090086 | G / A | 0.47 |
|  | rs915889 | 6 | 26095291 | G / A | 0.07 |
|  | rs2854030 | 6 | 26091118 | G / A | 0.29 |
|  | rs1800855 | 0 |  |  | Failed |
| *GHRL* (chr 3) | rs171336 | 6 | 10300749 | C / A | 0.36 |
|  | rs35683 | 6 | 10303249 | C / A | 0.48 |
|  | rs11718213 | 6 | 10300949 | A / C | 0.10 |
|  | rs35684 ^c^ | 5 | 10301685 | A / G | 0.28 |
|  | rs26802 | 6 | 10307364 | A / C | 0.33 |
|  | rs1617161 | 6 | 10311852 | G / A | 0.11 |
|  | rs27647 | 6 | 10307467 | A / G | 0.40 |
|  | rs17032621 | 6 | 10300637 | A / G | 0.14 |
|  | rs1629816 ^d^ | 2 | 10311290 | G / A | 0.38 |
|  | rs10490815 | 6 | 10310144 | A / G | 0.29 |
|  | rs3755777 | 6 | 10308363 | G / C | 0.25 |
|  | rs2619507 | 6 | 10310785 | A / G | 0.16 |
|  | rs4684677 | 0 |  |  | Failed |
| *GLP-1* (chr 2) | rs3761656 | 6 | 162719480 | A / C | 0.08 |
|  | rs13416088 | 6 | 162705904 | G / A | 0.21 |
|  | rs1990761 | 0 |  |  | Failed |
| *5-HT_1A_* (chr 5) | rs1423691 | 6 | 63287417 | A / G | 0.50 |
| *IL-6* (chr 7) | rs2069827 ^e^ | 2 | 22731980 | C / A | 0.09 |
|  | rs2069840 | 6 | 22735096 | G / C | 0.34 |
|  | rs12700386 | 6 | 22729533 | G / C | 0.19 |
|  | rs10242595 | 6 | 22740755 | G / A | 0.32 |
|  | rs2069861 | 6 | 22738178 | G / A | 0.09 |
|  | rs1800795 | 6 | 22733169 | C / G | 0.41 |
|  | rs2069837 | 6 | 22734551 | A / G | 0.08 |
| *LEP* (chr 7) | rs11763517 | 6 | 127677297 | A / G | 0.49 |
|  | rs3828942 ^f^ | 3 | 127681540 | G / A | 0.45 |
|  | rs2071045 | 6 | 127680215 | A / G | 0.24 |
|  | rs2278815 | 6 | 127669086 | A / G | 0.43 |
|  | rs11760956 | 6 | 127678322 | G / A | 0.37 |
|  | rs7788818 | 6 | 127681119 | G / A | 0.06 |
| *LEPR* (chr 1) | rs1137101 ^g^ | 2 | 65831100 | A / G | 0.46 |
|  | rs1022981 | 6 | 65772621 | A / G | 0.25 |
|  | rs1137100 | 6 | 65809028 | A / G | 0.24 |
|  | rs7516341 | 6 | 65860730 | A / G | 0.37 |
|  | rs1892534 | 6 | 65878531 | G / A | 0.38 |
|  | rs1892535 | 6 | 65869768 | G / A | 0.18 |
|  | rs3790426 | 6 | 65815606 | C / A | 0.24 |
|  | rs9436746 | 6 | 65681060 | C / A | 0.40 |
|  | rs970467 | 6 | 65679349 | G / A | 0.11 |
|  | rs1045895 | 6 | 65670568 | G / A | 0.40 |
|  | rs10493380 | 6 | 65818704 | A / C | 0.19 |
|  | rs6588147 | 6 | 65708081 | A / G | 0.32 |
|  | rs2025805 | 6 | 65722465 | G / A | 0.47 |
|  | rs3790433 | 6 | 65666929 | G / A | 0.26 |
|  | rs1171279 | 6 | 65761080 | G / A | 0.27 |
|  | rs1171267 ^h^ | 4 | 65776441 | C / A | 0.34 |
|  | rs11208659 | 6 | 65751867 | A / G | 0.10 |
|  | rs6704167 ^i^ | 5 | 65710467 | A / T | 0.45 |
|  | rs1171278 ^j^ | 3 | 65760733 | G / A | 0.18 |
|  | rs12409877 | 6 | 65716459 | G / A | 0.39 |
|  | rs8179183 | 6 | 65848539 | G / C | 0.18 |
|  | rs6662904 | 6 | 65770327 | G / A | 0.48 |
|  | rs3806318 ^c^ | 5 | 65657944 | A / G | 0.28 |
|  | rs4655537 | 6 | 65831388 | G / A | 0.36 |
|  | rs9436301 | 6 | 65668514 | A / G | 0.24 |
|  | rs4655802 | 6 | 65660818 | A / G | 0.41 |
|  | rs12145690 | 6 | 65659600 | A / C | 0.45 |
|  | rs1887285 | 6 | 65670334 | A / G | 0.09 |
|  | rs9436740 | 6 | 65664488 | T / A | 0.28 |
|  | rs11585329 | 6 | 65846401 | C / A | 0.15 |
|  | rs3762274 ^k^ | 5 | 65836700 | A / G | 0.39 |
|  | rs6673324 | 6 | 65803650 | A / G | 0.49 |
|  | rs10158579 | 6 | 65722643 | A / G | 0.13 |
|  | rs9436297 | 6 | 65661441 | A / G | 0.14 |
|  | rs6672331 | 6 | 65748434 | G / C | 0.03 |
|  | rs9436302 | 0 |  |  | Failed |
| *MC4R* (chr 18) | rs11872992 | 6 | 56191566 | G / A | 0.13 |
|  | rs8093815 | 6 | 56187482 | G / A | 0.31 |
|  | rs1943226 | 6 | 56186183 | A / C | 0.10 |
|  | rs1943220 | 0 |  |  | Failed |
|  | rs17066846 | 0 |  |  | Failed |
| *mTOR* (chr 1) | rs1770345 ^a^ | 2 | 11137166 | A / C | 0.47 |
|  | rs1074078 | 6 | 11249374 | G / A | 0.33 |
|  | rs12732063 | 6 | 11113818 | G / A | 0.05 |
|  | rs1057079 ^b^ | 3 | 11127644 | A / G | 0.26 |
| *NMB* (chr 15) | rs7180849 ^c^ | 5 | 82996662 | G / A | 0.17 |
|  | rs2292462 | 6 | 83001757 | A / C | 0.47 |
|  | rs17598561 ^l^ | 4 | 82999609 | G / A | 0.06 |
|  | rs1051168 | 6 | 83001523 | C / A | 0.29 |
|  | rs309430 | 0 |  |  | Failed |
| *NPY* (chr 7) | rs16472 | 6 | 24300593 | G / A | 0.09 |
|  | rs5574 | 6 | 24295657 | G / A | 0.47 |
|  | rs9785023 | 6 | 24291533 | A / G | 0.50 |
|  | rs16135 ^c^ | 5 | 24294444 | G / A | 0.07 |
|  | rs16141 ^c^ | 5 | 24291283 | A / C | 0.49 |
|  | rs3025118 | 6 | 24294201 | C / A | 0.04 |
|  | rs16148 ^c^ | 5 | 24288862 | A / G | 0.34 |
|  | rs12700524 | 6 | 24287938 | A / G | 0.14 |
|  | rs16142 | 0 |  |  | Failed |
|  | rs2023890 | 0 |  |  | Failed |
| *NUCB2* (chr 11) | rs214075 | 6 | 17256992 | C / A | 0.41 |
|  | rs10832763 ^c^ | 5 | 17307778 | A / G | 0.36 |
|  | rs7127347 | 6 | 17257419 | A / C | 0.13 |
|  | rs2634462 | 6 | 17295702 | G / A | 0.27 |
|  | rs10741725 | 6 | 17277372 | C / A | 0.46 |
|  | rs214105 | 6 | 17270096 | A / G | 0.28 |
|  | rs757081 | 6 | 17308258 | C / G | 0.32 |
|  | rs12419530 | 6 | 17262798 | A / G | 0.04 |
|  | rs214086 | 6 | 17255041 | G / C | 0.42 |
|  | rs214082 | 6 | 17255965 | G / A | 0.41 |
|  | rs10766383 | 6 | 17286373 | C / A | 0.28 |
|  | rs1330 | 6 | 17272604 | G / A | 0.33 |
| *POMC* (chr 2) | rs6713532 | 6 | 25238336 | A / G | 0.23 |
|  | rs934778 | 6 | 25242727 | A / G | 0.30 |
|  | rs1866146 | 6 | 25234076 | A / G | 0.34 |
|  | rs6545975 | 6 | 25238988 | A / G | 0.39 |
|  | rs3769671 | 6 | 25243656 | A / C | 0.03 |
|  | rs6719226 | 6 | 25249515 | G / C | 0.04 |
|  | rs7565877 | 6 | 25239567 | A / G | 0.11 |
|  | rs7565427 | 6 | 25239141 | G / A | 0.13 |
|  | rs6734859 | 6 | 25233411 | G / A | 0.13 |
| *PYY* (chr 17) | rs3744419 | 6 | 39438166 | G / A | 0.20 |
|  | rs1859223 | 6 | 39435090 | G / C | 0.16 |
|  | rs8079623 | 6 | 39382855 | G / C | 0.11 |
|  | rs1058046 | 6 | 39386056 | G / C | 0.33 |
|  | rs1662754 | 6 | 39381050 | A / T | 0.44 |
|  | rs9907468 | 6 | 39407614 | G / A | 0.10 |
|  | rs1618809 | 6 | 39421450 | G / A | 0.37 |
|  | rs1642598 | 0 |  |  | Failed |

5-HT_1A_: Serotonin receptor gene; MAF: Minor Allele Frequency

^1^ Number of study centers for which the SNP information was available, 0 means either genotyping failed or the SNP did not pass the predefined quality control.

^a^. Available in Italy and the UK. ^b^. Available in Italy, Germany and Denmark. ^c^. Available in 5 center in 4 countries except for the UK. ^d^. Available in Italy and Germany. ^e^. Available in Italy and Denmark. ^f^. Available in The Netherlands (2 centers) and Germany. ^g^. Available in the 2 center in The Netherlands. ^h^. Available in Italy, The Netherlands (2 centers) and Denmark. ^i^. Available in 5 center in 4 countries except for Germany. ^j^. Available in Italy, UK and Germany. ^k^. Available in 5 center in 4 countries except for Denmark. ^l^. Available in 4 countries except for The Netherlands.
