# Supplementary material for: Genetic Polymorphisms in the Hypothalamic Pathway in Relation to Subsequent Weight Change – The DiOGenes Study
Source: PLoS One. 2011 Feb 24;6(2):e17436. doi: 10.1371/journal.pone.0017436 (PMC3044761; doi:10.1371/journal.pone.0017436)
Supplement: Table S2 — Cross-sectional associations of 123 single nucleotide polymorphisms (SNPs) from the hypothalamic pathway with baseline body mass index (BMI, kg/m2). (DOC) [file pone.0017436.s002.doc]

**Table S2: Cross-sectional associations of 123 single nucleotide polymorphisms (SNPs) from the hypothalamic pathway with baseline body mass index (BMI, kg/m^2^)^1^.**

| **Genes** | **SNPs** | **Major allele/ minor allele** | **MAF** | **1 minor allele** | | | | |
| --- | --- | --- | --- | --- | --- | --- | --- | --- |
|  |  |  |  | **Random subcohort** | | **Cases** | **Noncases** | ***P* value^2^** |
|  |  |  |  | **(n = 6,566)** | | **(n= 5,584)** | **(n= 5,507)** |  |
|  |  |  |  | **β ± SE** | ***P* value** | **β ± SE** | **β ± SE** |  |
| *CCK* | rs10460960 | A/G | 11 | 0.03 ± 0.11 | 0.81 | -0.04 ± 0.12 | 0.06 ± 0.12 | 0.62 |
| *CCK* | rs10865918 | A/C | 38 | -0.03 ± 0.06 | 0.59 | 0.07 ± 0.08 | -0.02 ± 0.07 | 0.36 |
| *CCK* | rs11129949 | A/C | 12 | 0.11 ± 0.13 | 0.41 | -0.03 ± 0.12 | 0.16 ± 0.11 | 0.19 |
| *CCK* | rs11571842 | G/A | 49 | -0.005 ± 0.06 | 0.93 | 0.04 ± 0.07 | 0.02 ± 0.07 | 0.65 |
| *CCK* | rs747455 | G/A | 24 | 0.02 ± 0.07 | 0.82 | -0.03 ± 0.11 | -0.004 ± 0.08 | 0.79 |
| *CCK* | rs7628795 | G/A | 41 | 0.07 ± 0.06 | 0.25 | -0.0008 ± 0.09 | 0.08 ± 0.07 | 0.50 |
| *CCK* | rs8192472 | G/A | 38 | 0.02 ± 0.06 | 0.79 | -0.01 ± 0.0.08 | 0.003 ± 0.07 | 0.86 |
| *CCK* | rs9311317 | A/G | 25 | 0.08 ± 0.10 | 0.47 | -0.17 ± 0.09 | 0.12 ± 0.10 | **0.04** |
| *CCKAR* | rs1573596 | G/A | 47 | -0.006 ± 0.06 | 0.91 | -0.19 ± 0.07 | 0.04 ± 0.07 | **0.02** |
| *CCKAR* | rs2000978 | A/G | 17 | -0.06 ± 0.08 | 0.50 | -0.27 ± 0.09 | -0.04 ± 0.09 | 0.08 |
| *CCKAR* | rs2854030 | G/A | 29 | 0.01 ± 0.07 | 0.84 | -0.09 ± 0.08 | 0.06 ± 0.07 | 0.16 |
| *CCKAR* | rs7665027 | A/G | 15 | 0.05 ± 0.11 | 0.64 | -0.05 ± 0.13 | 0.06 ± 0.09 | 0.35 |
| *CCKAR* | rs915889 | G/A | 7 | -0.10 ± 0.12 | 0.38 | -0.006 ± 0.15 | -0.02 ± 0.12 | 0.92 |
| *mTOR* | rs1057079 | A/G | 26 | 0.16 ± 0.09 | 0.09 | 0.25 ± 0.17 | 0.18 ± 0.09 | 0.80 |
| *mTOR* | rs1074078 | G/A | 33 | -0.02 ± 0.06 | 0.74 | 0.17 ± 0.08 | 0.04 ± 0.07 | 0.26 |
| *mTOR* | rs12732063 | G/A | 5 | -0.14 ± 0.16 | 0.38 | 0.07 ± 0.22 | -0.25 ± 0.15 | 0.26 |
| *mTOR* | rs1770345 | A/C | 47 | 0.08 ± 0.09 | 0.42 | 0.04 ± 0.11 | 0.03 ± 0.10 | 0.92 |
| *GLP-1* | rs13416088 | G/A | 21 | -0.02 ± 0.09 | 0.87 | -0.09 ± 0.09 | 0.04 ± 0.14 | 0.32 |
| *GLP-1* | rs3761656 | A/C | 8 | -0.06 ± 0.13 | 0.64 | -0.08 ± 0.14 | -0.001 ± 0.12 | 0.74 |
| *GHRL* | rs10490815 | A/G | 29 | 0.07 ± 0.08 | 0.36 | 0.13 ± 0.08 | 0.01 ± 0.08 | 0.22 |
| *GHRL* | rs11718213 | A/C | 10 | 0.09 ± 0.10 | 0.41 | -0.09 ± 0.12 | 0.13 ± 0.11 | 0.17 |
| *GHRL* | rs1617161 | G/A | 11 | 0.06 ± 0.11 | 0.561 | -0.16 ± 0.11 | 0.09 ± 0.11 | 0.10 |
| *GHRL* | rs1629816 | G/A | 38 | 0.01 ± 0.09 | 0.92 | 0.02 ± 0.12 | -0.04 ± 0.10 | 0.65 |
| *GHRL* | rs17032621 | A/G | 14 | -0.05 ± 0.09 | 0.58 | 0.16 ± 0.11 | -0.11 ± 0.09 | **0.04** |
| *GHRL* | rs171336 | C/A | 36 | -0.03 ± 0.06 | 0.65 | -0.14 ± 0.08 | -0.03 ± 0.07 | 0.23 |
| *GHRL* | rs2619507 | A/G | 16 | -0.006 ± 0.09 | 0.95 | -0.11 ± 0.10 | 0.03 ± 0.09 | 0.32 |
| *GHRL* | rs26802 | A/C | 33 | -0.02 ± 0.08 | 0.79 | 0.01 ± 0.08 | -0.02 ± 0.07 | 0.68 |
| *GHRL* | rs27647 | A/G | 40 | 0.05 ± 0.06 | 0.45 | -0.04 ± 0.08 | 0.05 ± 0.07 | 0.35 |
| *GHRL* | rs35683 | C/A | 48 | 0.04 ± 0.07 | 0.57 | -0.09 ± 0.08 | 0.05 ± 0.08 | 0.21 |
| *GHRL* | rs35684 | A/G | 28 | -0.09 ± 0.08 | 0.27 | 0.06 ± 0.09 | -0.08 ± 0.09 | 0.23 |
| *GHRL* | rs3755777 | G/C | 25 | 0.04 ± 0.07 | 0.62 | 0.09 ± 0.09 | -0.02 ± 0.08 | 0.28 |
| *5-HT_1A_* | rs1423691 | A/G | 50 | -0.05 ± 0.07 | 0.52 | -0.15 ± 0.09 | -0.03 ± 0.07 | 0.24 |
| *I-L6* | rs10242595 | G/A | 32 | 0.10 ± 0.07 | 0.11 | 0.06 ± 0.08 | 0.07 ± 0.09 | 0.96 |
| *IL-6* | rs12700386 | G/C | 19 | -0.02 ± 0.08 | 0.84 | -0.12 ± 0.09 | -0.03 ± 0.08 | 0.41 |
| *IL-6* | rs1800795 | C/G | 41 | -0.02 ± 0.06 | 0.71 | -0.003 ± 0.08 | 0.03 ± 0.07 | 0.77 |
| *IL-6* | rs2069827 | C/A | 9 | 0.22 ± 0.17 | 0.20 | 0.002 ± 0.21 | 0.18 ± 0.18 | 0.56 |
| *IL-6* | rs2069837 | A/G | 8 | 0.03 ± 0.12 | 0.78 | 0.14 ± 0.16 | 0.04 ± 0.12 | 0.70 |
| *IL-6* | rs2069840 | G/C | 34 | -0.09 ± 0.07 | 0.21 | -0.07 ± 0.08 | -0.11 ± 0.07 | 0.72 |
| *IL-6* | rs2069861 | G/A | 9 | 0.17 ± 0.11 | 0.11 | 0.11 ± 0.13 | 0.14 ± 0.11 | 0.98 |
| *LEP* | rs11760956 | G/A | 37 | -0.06 ± 0.06 | 0.34 | -0.05 ± 0.08 | -0.06 ± 0.07 | 0.94 |
| *LEP* | rs11763517 | A/G | 49 | 0.009 ± 0.06 | 0.88 | -0.11 ± 0.11 | 0.009 ± 0.07 | 0.32 |
| *LEP* | rs2071045 | A/G | 24 | 0.12 ± 0.08 | 0.15 | -0.009 ± 0.12 | 0.12 ± 0.08 | 0.30 |
| *LEP* | rs2278815 | A/G | 43 | -0.14 ± 0.06 | **0.03** | -0.06 ± 0.08 | -0.12 ± 0.08 | 0.56 |
| *LEP* | rs3828942 | G/A | 45 | 0.07 ± 0.09 | 0.45 | 0.14 ± 0.12 | 0.02 ± 0.10 | 0.56 |
| *LEP* | rs7788818 | G/A | 6 | -0.18 ± 0.13 | 0.17 | -0.02 ± 0.25 | -0.08 ± 0.15 | 0.92 |
| *LEPR* | rs10158579 | A/G | 13 | -0.006 ± 0.09 | 0.95 | 0.06 ± 0.11 | -0.05 ± 0.09 | 0.38 |
| *LEPR* | rs1022981 | A/G | 25 | -0.03 ± 0.07 | 0.63 | -0.05 ± 0.17 | -0.05 ± 0.07 | 0.97 |
| *LEPR* | rs1045895 | G/A | 40 | -0.008 ± 0.06 | 0.90 | -0.006 ± 0.13 | 0.03 ± 0.07 | 0.94 |
| *LEPR* | rs10493380 | A/C | 19 | -0.11 ± 0.08 | 0.15 | -0.21 ± 0.09 | -0.04 ± 0.08 | 0.15 |
| *LEPR* | rs11208659 | A/G | 10 | 0.15 ± 0.13 | 0.24 | 0.20 ± 0.18 | 0.16 ± 0.11 | 0.83 |
| *LEPR* | rs1137100 | A/G | 24 | -0.05 ± 0.07 | 0.46 | -0.07 ± 0.18 | -0.07 ± 0.07 | 0.95 |
| *LEPR* | rs1137101 | A/G | 46 | -0.15 ± 0.13 | 0.26 | -0.08 ± 0.21 | -0.07 ± 0.14 | 0.91 |
| *LEPR* | rs11585329 | C/A | 15 | -0.02 ± 0.11 | 0.87 | -0.08 ± 0.10 | -0.02 ± 0.14 | 0.82 |
| *LEPR* | rs1171267 | C/A | 34 | 0.03 ± 0.085 | 0.74 | -0.08 ± 0.10 | 0.09 ± 0.09 | 0.15 |
| *LEPR* | rs1171278 | G/A | 18 | -0.02 ± 0.09 | 0.84 | 0.02 ± 0.12 | -0.05 ± 0.11 | 0.72 |
| *LEPR* | rs1171279 | G/A | 27 | 0.05 ± 0.07 | 0.51 | 0.11 ± 0.12 | 0.03 ± 0.07 | 0.56 |
| *LEPR* | rs12145690 | A/C | 45 | 0.01 ± 0.06 | 0.83 | -0.04 ± 0.07 | -0.02 ± 0.07 | 0.82 |
| *LEPR* | rs12409877 | G/A | 39 | 0.05 ± 0.06 | 0.40 | 0.03 ± 0.11 | 0.02 ± 0.07 | 0.92 |
| *LEPR* | rs1887285 | A/G | 9 | -0.20 ± 0.10 | 0.052 | -0.25 ± 0.19 | -0.16 ± 0.11 | 0.66 |
| *LEPR* | rs1892534 | G/A | 38 | -0.11 ± 0.08 | 0.17 | -0.15 ± 0.11 | -0.05 ± 0.07 | 0.40 |
| *LEPR* | rs1892535 | G/A | 18 | -0.05 ± 0.10 | 0.66 | 0.03 ± 0.19 | -0.04 ± 0.10 | 0.77 |
| *LEPR* | rs2025805 | G/A | 47 | -0.06 ± 0.06 | 0.33 | -0.06 ± 0.12 | -0.01 ± 0.06 | 0.73 |
| *LEPR* | rs3762274 | A/G | 39 | 0.09 ± 0.08 | 0.26 | 0.21 ± 0.09 | 0.06 ± 0.08 | 0.30 |
| *LEPR* | rs3790426 | C/A | 24 | 0.18 ± 0.07 | **0.01** | 0.26 ± 0.12 | 0.14 ± 0.08 | 0.36 |
| *LEPR* | rs3790433 | G/A | 26 | -0.02 ± 0.07 | 0.83 | 0.04 ± 0.09 | -0.0008 ± 0.07 | 0.71 |
| *LEPR* | rs3806318 | A/G | 28 | -0.03 ± 0.08 | 0.71 | 0.05 ± 0.14 | -0.06 ± 0.08 | 0.58 |
| *LEPR* | rs4655537 | G/A | 36 | 0.03 ± 0.06 | 0.60 | 0.13 ± 0.08 | -0.02 ± 0.07 | 0.14 |
| *LEPR* | rs4655802 | A/G | 41 | 0.05 ± 0.06 | 0.39 | -0.09 ± 0.08 | -0.02 ± 0.07 | 0.57 |
| *LEPR* | rs6588147 | A/G | 32 | 0.01 ± 0.07 | 0.83 | -0.03 ± 0.13 | -0.02 ± 0.07 | 0.95 |
| *LEPR* | rs6662904 | G/A | 48 | 0.009 ± 0.06 | 0.88 | -0.04 ± 0.09 | 0.04 ± 0.06 | 0.37 |
| *LEPR* | rs6672331 | G/C | 3 | -0.14 ± 0.19 | 0.46 | 0.006 ± 0.22 | -0.24 ± 0.19 | 0.42 |
| *LEPR* | rs6673324 | A/G | 49 | -0.09 ± 0.06 | 0.10 | -0.16 ± 0.09 | -0.08 ± 0.06 | 0.33 |
| *LEPR* | rs6704167 | A/T | 45 | -0.04 ± 0.07 | 0.59 | -0.03 ± 0.14 | -0.01 ± 0.07 | 0.92 |
| *LEPR* | rs7516341 | A/G | 37 | -0.09 ± 0.08 | 0.22 | -0.15 ± 0.11 | -0.04 ± 0.07 | 0.35 |
| *LEPR* | rs8179183 | G/C | 18 | -0.16 ± 0.08 | 0.050 | -0.35 ± 0.09 | -0.05 ± 0.08 | **0.02** |
| *LEPR* | rs9436297 | A/G | 14 | 0.07 ± 0.09 | 0.46 | 0.29 ± 0.11 | 0.06 ± 0.09 | 0.15 |
| *LEPR* | rs9436301 | A/G | 24 | -0.09 ± 0.07 | 0.17 | -0.009 ± 0.09 | -0.11 ± 0.07 | 0.37 |
| *LEPR* | rs9436740 | T/A | 28 | 0.07 ± 0.09 | 0.48 | -0.11 ± 0.13 | 0.09 ± 0.13 | 0.48 |
| *LEPR* | rs9436746 | C/A | 40 | 0.04 ± 0.06 | 0.49 | -0.02 ± 0.13 | 0.03 ± 0.07 | 0.71 |
| *LEPR* | rs970467 | G/A | 11 | 0.04 ± 0.12 | 0.74 | 0.18 ± 0.12 | 0.01 ± 0.14 | 0.21 |
| *MC4R* | rs11872992 | G/A | 13 | -0.11 ± 0.09 | 0.24 | 0.03 ± 0.11 | -0.06 ± 0.11 | 0.64 |
| *MC4R* | rs1943226 | A/C | 10 | 0.12 ± 0.09 | 0.23 | 0.22 ± 0.12 | 0.14 ± 0.11 | 0.58 |
| *MC4R* | rs8093815 | G/A | 31 | 0.02 ± 0.07 | 0.78 | 0.01 ± 0.09 | -0.02 ± 0.07 | 0.82 |
| *NMB* | rs1051168 | C/A | 29 | -0.12 ± 0.09 | 0.18 | -0.07 ± 0.08 | -0.13 ± 0.08 | 0.65 |
| *NMB* | rs17598561 | G/A | 6 | -0.07 ± 0.15 | 0.63 | 0.13 ± 0.33 | -0.06 ± 0.18 | 0.58 |
| *NMB* | rs2292462 | A/C | 47 | -0.03 ± 0.06 | 0.58 | -0.08 ± 0.07 | -0.04 ± 0.07 | 0.57 |
| *NMB* | rs7180849 | G/A | 17 | 0.03 ± 0.13 | 0.80 | -0.09 ± 0.11 | 0.03 ± 0.15 | 0.54 |
| *NPY* | rs12700524 | A/G | 14 | -0.03 ± 0.11 | 0.81 | 0.18 ± 0.11 | -0.09 ± 0.11 | 0.14 |
| *NPY* | rs16135 | G/A | 7 | 0.03 ± 0.13 | 0.83 | 0.18 ± 0.27 | -0.02 ± 0.14 | 0.40 |
| *NPY* | rs16141 | A/C | 49 | -0.03 ± 0.07 | 0.70 | -0.12 ± 0.09 | -0.0006 ± 0.08 | 0.26 |
| *NPY* | rs16148 | A/G | 34 | -0.004 ± 0.07 | 0.95 | -0.04 ± 0.09 | 0.007 ± 0.08 | 0.79 |
| *NPY* | rs16472 | G/A | 9 | 0.05 ± 0.11 | 0.65 | 0.32 ± 0.23 | 0.008 ± 0.117 | 0.11 |
| *NPY* | rs3025118 | C/A | 4 | 0.16 ± 0.16 | 0.31 | -0.04 ± 0.20 | 0.17 ± 0.17 | 0.47 |
| *NPY* | rs5574 | G/A | 47 | -0.02 ± 0.07 | 0.71 | -0.14 ± 0.08 | 0.005 ± 0.08 | 0.14 |
| *NPY* | rs9785023 | G/A | 50 | -0.007 ± 0.07 | 0.92 | 0.19 ± 0.08 | -0.03 ± 0.07 | **0.03** |
| *NUCB2* | rs10741725 | C/A | 46 | 0.03 ± 0.07 | 0.66 | -0.13 ± 0.08 | 0.04 ± 0.07 | 0.09 |
| *NUCB2* | rs10766383 | C/A | 28 | 0.03 ± 0.07 | 0.65 | 0.007 ± 0.08 | 0.03 ± 0.07 | 0.89 |
| *NUCB2* | rs10832763 | A/G | 36 | 0.16 ± 0.07 | **0.03** | 0.085 ± 0.09 | 0.16 ± 0.08 | 0.45 |
| *NUCB2* | rs12419530 | A/G | 4 | 0.16 ± 0.19 | 0.38 | -0.14 ± 0.19 | 0.05 ± 0.17 | 0.56 |
| *NUCB2* | rs1330 | G/A | 33 | -0.05 ± 0.07 | 0.43 | -0.17 ± 0.08 | -0.04 ± 0.09 | 0.25 |
| *NUCB2* | rs214075 | C/A | 41 | 0.05 ± 0.09 | 0.58 | -0.12 ± 0.08 | 0.05 ± 0.11 | 0.15 |
| *NUCB2* | rs214082 | G/A | 41 | -0.04 ± 0.06 | 0.56 | -0.02 ± 0.08 | -0.006 ± 0.09 | 0.88 |
| *NUCB2* | rs214086 | G/C | 42 | -0.08 ± 0.09 | 0.42 | -0.02 ± 0.08 | -0.12 ± 0.12 | 0.55 |
| *NUCB2* | rs214105 | A/G | 28 | -0.04 ± 0.09 | 0.65 | -0.19 ± 0.08 | -0.04 ± 0.113 | 0.29 |
| *NUCB2* | rs2634462 | G/A | 27 | -0.07 ± 0.08 | 0.39 | 0.15 ± 0.08 | -0.08 ± 0.09 | **0.03** |
| *NUCB2* | rs7127347 | A/C | 13 | 0.19 ± 0.12 | 0.11 | 0.07 ± 0.11 | 0.14 ± 0.09 | 0.71 |
| *NUCB2* | rs757081 | C/G | 32 | 0.01 ± 0.07 | 0.86 | -0.16 ± 0.08 | 0.009 ± 0.08 | 0.11 |
| *POMC* | rs1866146 | A/G | 34 | 0.08 ± 0.06 | 0.22 | -0.11 ± 0.08 | 0.04 ± 0.07 | 0.13 |
| *POMC* | rs3769671 | A/C | 3 | -0.23 ± 0.22 | 0.31 | 0.07 ± 0.22 | -0.18 ± 0.21 | 0.46 |
| *POMC* | rs6545975 | A/G | 39 | -0.02 ± 0.06 | 0.79 | 0.08 ± 0.08 | -0.009 ± 0.07 | 0.34 |
| *POMC* | rs6713532 | A/G | 23 | 0.08 ± 0.08 | 0.29 | -0.02 ± 0.09 | 0.03 ± 0.08 | 0.89 |
| *POMC* | rs6719226 | G/C | 4 | -0.06 ± 0.18 | 0.75 | 0.21 ± 0.18 | -0.18 ± 0.19 | 0.15 |
| *POMC* | rs6734859 | G/A | 13 | 0.009 ± 0.11 | 0.93 | -0.15 ± 0.11 | -0.04 ± 0.12 | 0.54 |
| *POMC* | rs7565427 | G/A | 13 | 0.01 ± 0.11 | 0.91 | -0.13 ± 0.11 | -0.04 ± 0.12 | 0.71 |
| *POMC* | rs7565877 | A/G | 11 | -0.12 ± 0.13 | 0.37 | 0.05 ± 0.12 | -0.16 ± 0.14 | 0.33 |
| *POMC* | rs934778 | A/G | 30 | -0.02 ± 0.07 | 0.72 | 0.17 ± 0.08 | -0.01 ± 0.07 | 0.10 |
| *PYY* | rs1058046 | G/C | 33 | 0.03 ± 0.08 | 0.73 | 0.06 ± 0.08 | 0.05 ± 0.09 | 0.87 |
| *PYY* | rs1618809 | G/A | 37 | 0.01 ± 0.11 | 0.92 | -0.02 ± 0.08 | 0.04 ± 0.13 | 0.73 |
| *PYY* | rs1662754 | A/T | 44 | 0.02 ± 0.07 | 0.78 | 0.02 ± 0.09 | 0.02 ± 0.09 | 0.85 |
| *PYY* | rs1859223 | G/C | 16 | -0.05 ± 0.10 | 0.59 | 0.05 ± 0.11 | -0.01 ± 0.11 | 0.69 |
| *PYY* | rs3744419 | G/A | 20 | 0.07 ± 0.12 | 0.52 | -0.16 ± 0.09 | 0.10 ± 0.12 | 0.10 |
| *PYY* | rs8079623 | G/C | 11 | 0.01 ± 0.09 | 0.91 | -0.09 ± 0.15 | -0.05 ± 0.14 | 0.92 |
| *PYY* | rs9907468 | G/A | 10 | -0.14 ± 0.11 | 0.20 | 0.16 ± 0.13 | -0.11 ± 0.11 | 0.08 |

MAF: Minor Allele Frequency; SE: Standard Error

^1^ Values presented are the overall meta-analyzed regression coefficients and *P* values.

^2^ *P* values are for the differences in baseline BMI between cases and non-cases.
