# Supplementary material for: Genetic Polymorphisms in the Hypothalamic Pathway in Relation to Subsequent Weight Change – The DiOGenes Study
Source: PLoS One. 2011 Feb 24;6(2):e17436. doi: 10.1371/journal.pone.0017436 (PMC3044761; doi:10.1371/journal.pone.0017436)
Supplement: Table S3 — Association of 123 single nucleotide polymorphisms (SNPs) from the hypothalamic pathway with the risk of being a ‘weight gainer’ in case - noncase analysis (n = 11,091). (DOC) [file pone.0017436.s003.doc]

**Table S3. Association of 123 single nucleotide polymorphisms (SNPs) from the hypothalamic pathway with the risk of being a ‘weight gainer’ in case - noncase analysis (n = 11,091)^1^.**

| **Genes** | **SNPs** | **Major allele/ minor allele** | **MAF** | **1 minor allele** | | |
| --- | --- | --- | --- | --- | --- | --- |
|  |  |  |  | **Odds ratio** | **95% CI** | ***P* value** |
| *CCK* | rs10460960 | A/G | 11 | 1.03 | 0.95-1.12 | 0.43 |
| *CCK* | rs10865918 | A/C | 38 | 1.05 | 0.99-1.11 | 0.08 |
| *CCK* | rs11129949 | A/C | 12 | 1.03 | 0.94-1.14 | 0.50 |
| *CCK* | rs11571842 | G/A | 49 | 1.06 | 1.00-1.12 | **0.03** |
| *CCK* | rs747455 | G/A | 24 | 0.95 | 0.90-1.01 | 0.14 |
| *CCK* | rs7628795 | G/A | 41 | 0.99 | 0.93-1.04 | 0.61 |
| *CCK* | rs8192472 | G/A | 38 | 0.98 | 0.92-1.03 | 0.42 |
| *CCK* | rs9311317 | A/G | 25 | 0.98 | 0.93-1.05 | 0.59 |
| *CCKAR* | rs1573596 | G/A | 47 | 0.99 | 0.95-1.05 | 0.93 |
| *CCKAR* | rs2000978 | A/G | 17 | 1.00 | 0.93-1.08 | 0.94 |
| *CCKAR* | rs2854030 | G/A | 29 | 0.99 | 0.94-1.06 | 0.99 |
| *CCKAR* | rs7665027 | A/G | 15 | 1.01 | 0.94-1.09 | 0.73 |
| *CCKAR* | rs915889 | G/A | 7 | 0.97 | 0.87-1.07 | 0.51 |
| *mTOR* | rs1057079 | A/G | 26 | 1.02 | 0.95-1.11 | 0.58 |
| *mTOR* | rs1074078 | G/A | 33 | 0.96 | 0.91-1.02 | 0.17 |
| *mTOR* | rs12732063 | G/A | 5 | 1.02 | 0.90-1.16 | 0.73 |
| *mTOR* | rs1770345 | A/C | 47 | 0.99 | 0.80-1.23 | 0.96 |
| *GLP-1* | rs13416088 | G/A | 21 | 1.01 | 0.94-1.09 | 0.77 |
| *GLP-1* | rs3761656 | A/C | 8 | 1.00 | 0.91-1.11 | 0.05 |
| *GHRL* | rs10490815 | A/G | 29 | 0.97 | 0.92-1.03 | 0.40 |
| *GHRL* | rs11718213 | A/C | 10 | 1.03 | 0.94-1.12 | 0.55 |
| *GHRL* | rs1617161 | G/A | 11 | 1.03 | 0.93-1.14 | 0.59 |
| *GHRL* | rs1629816 | G/A | 38 | 1.04 | 0.96-1.14 | 0.33 |
| *GHRL* | rs17032621 | A/G | 14 | 1.04 | 0.97-1.12 | 0.28 |
| *GHRL* | rs171336 | C/A | 36 | 0.98 | 0.93-1.04 | 0.53 |
| *GHRL* | rs2619507 | A/G | 16 | 1.02 | 0.94-1.11 | 0.66 |
| *GHRL* | rs26802 | A/C | 33 | 1.02 | 0.96-1.08 | 0.47 |
| *GHRL* | rs27647 | A/G | 40 | 0.99 | 0.93-1.04 | 0.61 |
| *GHRL* | rs35683 | C/A | 48 | 0.97 | 0.92-1.02 | 0.27 |
| *GHRL* | rs35684 | A/G | 28 | 1.02 | 0.93-1.11 | 0.69 |
| *GHRL* | rs3755777 | G/C | 25 | 0.99 | 0.94-1.06 | 0.88 |
| *5-HT_1A_* | rs1423691 | A/G | 50 | 1.02 | 0.97-1.08 | 0.44 |
| *IL-6* | rs10242595 | G/A | 32 | 0.98 | 0.92-1.03 | 0.40 |
| *IL-6* | rs12700386 | G/C | 19 | 1.01 | 0.94-1.08 | 0.86 |
| *IL-6* | rs1800795 | C/G | 41 | 1.01 | 0.95-1.06 | 0.82 |
| *IL-6* | rs2069827 | C/A | 9 | 0.97 | 0.84-1.12 | 0.66 |
| *IL-6* | rs2069837 | A/G | 8 | 0.94 | 0.85-1.03 | 0.20 |
| *IL-6* | rs2069840 | G/C | 34 | 1.03 | 0.97-1.09 | 0.34 |
| *IL-6* | rs2069861 | G/A | 9 | 1.04 | 0.95-1.13 | 0.43 |
| *LEP* | rs11760956 | G/A | 37 | 0.99 | 0.94-1.05 | 0.85 |
| *LEP* | rs11763517 | A/G | 49 | 0.99 | 0.95-1.05 | 0.88 |
| *LEP* | rs2071045 | A/G | 24 | 1.00 | 0.94-1.06 | 0.99 |
| *LEP* | rs2278815 | A/G | 43 | 1.02 | 0.97-1.08 | 0.42 |
| *LEP* | rs3828942 | G/A | 45 | 0.99 | 0.91-1.08 | 0.79 |
| *LEP* | rs7788818 | G/A | 6 | 1.03 | 0.87-1.22 | 0.72 |
| *LEPR* | rs10158579 | A/G | 13 | 1.05 | 0.97-1.13 | 0.23 |
| *LEPR* | rs1022981 | A/G | 25 | 1.05 | 0.98-1.11 | 0.14 |
| *LEPR* | rs1045895 | G/A | 40 | 0.98 | 0.92-1.04 | 0.50 |
| *LEPR* | rs10493380 | A/C | 19 | 0.96 | 0.90-1.03 | 0.25 |
| *LEPR* | rs11208659 | A/G | 10 | 0.96 | 0.83-1.10 | 0.52 |
| *LEPR* | rs1137100 | A/G | 24 | 1.03 | 0.97-1.10 | 0.27 |
| *LEPR* | rs1137101 | A/G | 46 | 1.01 | 0.89-1.14 | 0.90 |
| *LEPR* | rs11585329 | C/A | 15 | 1.02 | 0.95-1.10 | 0.54 |
| *LEPR* | rs1171267 | C/A | 34 | 0.98 | 0.91-1.06 | 0.63 |
| *LEPR* | rs1171278 | G/A | 18 | 1.09 | 0.99-1.19 | 0.07 |
| *LEPR* | rs1171279 | G/A | 27 | 1.01 | 0.95-1.07 | 0.86 |
| *LEPR* | rs12145690 | A/C | 45 | 1.03 | 0.98-1.09 | 0.23 |
| *LEPR* | rs12409877 | G/A | 39 | 1.01 | 0.94-1.08 | 0.82 |
| *LEPR* | rs1887285 | A/G | 9 | 0.99 | 0.91-1.09 | 0.92 |
| *LEPR* | rs1892534 | G/A | 38 | 0.98 | 0.93-1.04 | 0.58 |
| *LEPR* | rs1892535 | G/A | 18 | 1.00 | 0.94-1.07 | 0.93 |
| *LEPR* | rs2025805 | G/A | 47 | 0.97 | 0.91-1.03 | 0.37 |
| *LEPR* | rs3762274 | A/G | 39 | 0.99 | 0.94-1.06 | 0.89 |
| *LEPR* | rs3790426 | C/A | 24 | 0.97 | 0.88-1.08 | 0.57 |
| *LEPR* | rs3790433 | G/A | 26 | 0.99 | 0.93-1.05 | 0.63 |
| *LEPR* | rs3806318 | A/G | 28 | 1.02 | 0.95-1.09 | 0.60 |
| *LEPR* | rs4655537 | G/A | 36 | 1.02 | 0.96-1.07 | 0.59 |
| *LEPR* | rs4655802 | A/G | 41 | 0.96 | 0.91-1.02 | 0.17 |
| *LEPR* | rs6588147 | A/G | 32 | 1.04 | 0.99-1.10 | 0.15 |
| *LEPR* | rs6662904 | G/A | 48 | 0.96 | 0.91-1.01 | 0.13 |
| *LEPR* | rs6672331 | G/C | 3 | 0.99 | 0.85-1.15 | 0.89 |
| *LEPR* | rs6673324 | A/G | 49 | 0.99 | 0.92-1.07 | 0.86 |
| *LEPR* | rs6704167 | A/T | 45 | 0.97 | 0.91-1.03 | 0.37 |
| *LEPR* | rs7516341 | A/G | 37 | 0.99 | 0.93-1.04 | 0.61 |
| *LEPR* | rs8179183 | G/C | 18 | 0.97 | 0.90-1.04 | 0.33 |
| *LEPR* | rs9436297 | A/G | 14 | 0.99 | 0.92-1.08 | 0.97 |
| *LEPR* | rs9436301 | A/G | 24 | 0.99 | 0.93-1.05 | 0.75 |
| *LEPR* | rs9436740 | T/A | 28 | 0.99 | 0.93-1.05 | 0.77 |
| *LEPR* | rs9436746 | C/A | 40 | 1.03 | 0.97-1.11 | 0.34 |
| *LEPR* | rs970467 | G/A | 11 | 1.02 | 0.94-1.11 | 0.69 |
| *MC4R* | rs11872992 | G/A | 13 | 0.99 | 0.86-1.16 | 0.99 |
| *MC4R* | rs1943226 | A/C | 10 | 0.99 | 0.91-1.07 | 0.75 |
| *MC4R* | rs8093815 | G/A | 31 | 1.01 | 0.94-1.07 | 0.88 |
| *NMB* | rs1051168 | C/A | 29 | 1.04 | 0.98-1.10 | 0.19 |
| *NMB* | rs17598561 | G/A | 6 | 1.09 | 0.96-1.23 | 0.17 |
| *NMB* | rs2292462 | A/C | 47 | 1.05 | 0.99-1.10 | 0.09 |
| *NMB* | rs7180849 | G/A | 17 | 1.01 | 0.93-1.09 | 0.84 |
| *NPY* | rs12700524 | A/G | 14 | 1.01 | 0.89-1.16 | 0.85 |
| *NPY* | rs16135 | G/A | 7 | 1.10 | 0.98-1.23 | 0.09 |
| *NPY* | rs16141 | A/C | 49 | 1.03 | 0.97-1.09 | 0.36 |
| *NPY* | rs16148 | A/G | 34 | 0.99 | 0.92-1.06 | 0.73 |
| *NPY* | rs16472 | G/A | 9 | 0.98 | 0.89-1.08 | 0.73 |
| *NPY* | rs3025118 | C/A | 4 | 0.94 | 0.79-1.12 | 0.50 |
| *NPY* | rs5574 | G/A | 47 | 1.02 | 0.94-1.11 | 0.60 |
| *NPY* | rs9785023 | G/A | 50 | 0.96 | 0.89-1.03 | 0.22 |
| *NUCB2* | rs10741725 | C/A | 46 | 1.01 | 0.95-1.06 | 0.80 |
| *NUCB2* | rs10766383 | C/A | 28 | 1.02 | 0.95-1.09 | 0.61 |
| *NUCB2* | rs10832763 | A/G | 36 | 1.01 | 0.93-1.10 | 0.77 |
| *NUCB2* | rs12419530 | A/G | 4 | 0.98 | 0.07-0.86 | 0.81 |
| *NUCB2* | rs1330 | G/A | 33 | 1.00 | 0.95-1.06 | 0.97 |
| *NUCB2* | rs214075 | C/A | 41 | 1.03 | 0.98-1.09 | 0.30 |
| *NUCB2* | rs214082 | G/A | 41 | 1.02 | 0.96-1.07 | 0.57 |
| *NUCB2* | rs214086 | G/C | 42 | 0.99 | 0.94-1.05 | 0.88 |
| *NUCB2* | rs214105 | A/G | 28 | 1.02 | 0.96-1.08 | 0.47 |
| *NUCB2* | rs2634462 | G/A | 27 | 0.98 | 0.91-1.06 | 0.61 |
| *NUCB2* | rs7127347 | A/C | 13 | 1.00 | 0.93-1.08 | 0.95 |
| *NUCB2* | rs757081 | C/G | 32 | 1.02 | 0.96-1.08 | 0.48 |
| *POMC* | rs1866146 | A/G | 34 | 1.03 | 0.97-1.08 | 0.37 |
| *POMC* | rs3769671 | A/C | 3 | 1.02 | 0.82-1.27 | 0.84 |
| *POMC* | rs6545975 | A/G | 39 | 0.97 | 0.92-1.03 | 0.32 |
| *POMC* | rs6713532 | A/G | 23 | 1.02 | 0.96-1.08 | 0.54 |
| *POMC* | rs6719226 | G/C | 4 | 1.04 | 0.91-1.18 | 0.57 |
| *POMC* | rs6734859 | G/A | 13 | 1.05 | 0.98-1.14 | 0.18 |
| *POMC* | rs7565427 | G/A | 13 | 1.05 | 0.97-1.13 | 0.22 |
| *POMC* | rs7565877 | A/G | 11 | 1.06 | 0.97-1.15 | 0.20 |
| *POMC* | rs934778 | A/G | 30 | 0.96 | 0.91-1.02 | 0.17 |
| *PYY* | rs1058046 | G/C | 33 | 0.95 | 0.88-1.02 | 0.16 |
| *PYY* | rs1618809 | G/A | 37 | 0.97 | 0.91-1.02 | 0.21 |
| *PYY* | rs1662754 | A/T | 44 | 0.98 | 0.93-1.03 | 0.39 |
| *PYY* | rs1859223 | G/C | 16 | 0.91 | 0.85-0.98 | **0.01** |
| *PYY* | rs3744419 | G/A | 20 | 1.02 | 0.95-1.09 | 0.61 |
| *PYY* | rs8079623 | G/C | 11 | 1.08 | 0.99-1.17 | 0.09 |
| *PYY* | rs9907468 | G/A | 10 | 0.94 | 0.86-1.03 | 0.18 |

MAF: Minor Allele Frequency.

^1^ Values presented are the overall meta-analyzed regression coefficients and *P* values.
