# Supplementary material for: Genetic Polymorphisms in the Hypothalamic Pathway in Relation to Subsequent Weight Change – The DiOGenes Study
Source: PLoS One. 2011 Feb 24;6(2):e17436. doi: 10.1371/journal.pone.0017436 (PMC3044761; doi:10.1371/journal.pone.0017436)
Supplement: Table S5 — Association of 123 single nucleotide polymorphisms (SNPs) from the hypothalamic pathway with weight gain (g/year) in random subcohort analysis (n = 6,566). (DOC) [file pone.0017436.s005.doc]

**Table S5. Association of 123 single nucleotide polymorphisms (SNPs) from the hypothalamic pathway with weight gain (g/year) in random subcohort analysis (n = 6,566)^1^.**

| **Genes** | **SNPs** | **Major allele/ minor allele** | **MAF** | **1 minor allele** | | |
| --- | --- | --- | --- | --- | --- | --- |
|  |  |  |  | **β** | **SE** | ***P* value** |
| *CCK* | rs10460960 | A/G | 11 | 23 | 18.63 | 0.21 |
| *CCK* | rs10865918 | A/C | 38 | -2 | 11.95 | 0.86 |
| *CCK* | rs11129949 | A/C | 12 | 22 | 18.14 | 0.23 |
| *CCK* | rs11571842 | G/A | 49 | 7 | 11.61 | 0.52 |
| *CCK* | rs747455 | G/A | 24 | 0.3 | 17.11 | 0.99 |
| *CCK* | rs7628795 | G/A | 41 | -23 | 11.94 | 0.06 |
| *CCK* | rs8192472 | G/A | 38 | -5 | 12.00 | 0.66 |
| *CCK* | rs9311317 | A/G | 25 | -13 | 14.99 | 0.37 |
| *CCKAR* | rs1573596 | G/A | 47 | 1 | 11.61 | 0.91 |
| *CCKAR* | rs2000978 | A/G | 17 | 11 | 15.37 | 0.46 |
| *CCKAR* | rs2854030 | G/A | 29 | -0.1 | 12.68 | 0.99 |
| *CCKAR* | rs7665027 | A/G | 15 | 3 | 16.38 | 0.84 |
| *CCKAR* | rs915889 | G/A | 7 | -12 | 22.26 | 0.60 |
| *mTOR* | rs1057079 | A/G | 26 | 11 | 15.83 | 0.48 |
| *mTOR* | rs1074078 | G/A | 33 | -2 | 12.16 | 0.88 |
| *mTOR* | rs12732063 | G/A | 5 | 24 | 27.48 | 0.38 |
| *mTOR* | rs1770345 | A/C | 47 | -16 | 52.58 | 0.76 |
| *GLP1* | rs13416088 | G/A | 21 | -6 | 14.35 | 0.67 |
| *GLP1* | rs3761656 | A/C | 8 | -5 | 21.74 | 0.84 |
| *GHRL* | rs10490815 | A/G | 29 | -5 | 12.98 | 0.73 |
| *GHRL* | rs11718213 | A/C | 10 | 20 | 19.32 | 0.30 |
| *GHRL* | rs1617161 | G/A | 11 | 26 | 17.86 | 0.15 |
| *GHRL* | rs1629816 | G/A | 38 | 5 | 15.67 | 0.76 |
| *GHRL* | rs17032621 | A/G | 14 | 18 | 16.59 | 0.27 |
| *GHRL* | rs171336 | C/A | 36 | -11 | 12.15 | 0.36 |
| *GHRL* | rs2619507 | A/G | 16 | 22 | 15.62 | 0.16 |
| *GHRL* | rs26802 | A/C | 33 | 25 | 12.33 | **0.04** |
| *GHRL* | rs27647 | A/G | 40 | -13 | 11.82 | 0.29 |
| *GHRL* | rs35683 | C/A | 48 | -8 | 11.64 | 0.47 |
| *GHRL* | rs35684 | A/G | 28 | 1 | 15.77 | 0.94 |
| *GHRL* | rs3755777 | G/C | 25 | -4 | 13.54 | 0.80 |
| *5-HT_1A_* | rs1423691 | A/G | 50 | 6 | 17.66 | 0.72 |
| *IL-6* | rs10242595 | G/A | 32 | -16 | 14.01 | 0.25 |
| *IL-6* | rs12700386 | G/C | 19 | -8 | 17.25 | 0.63 |
| *IL-6* | rs1800795 | C/G | 41 | 2 | 11.77 | 0.89 |
| *IL-6* | rs2069827 | C/A | 9 | -1 | 30.28 | 0.96 |
| *IL-6* | rs2069837 | A/G | 8 | 1 | 20.27 | 0.96 |
| *IL-6* | rs2069840 | G/C | 34 | 5 | 12.28 | 0.69 |
| *IL-6* | rs2069861 | G/A | 9 | -0.2 | 20.86 | 0.99 |
| *LEP* | rs11760956 | G/A | 37 | -8 | 20.88 | 0.70 |
| *LEP* | rs11763517 | A/G | 49 | -6 | 17.33 | 0.73 |
| *LEP* | rs2071045 | A/G | 24 | 11 | 23.25 | 0.63 |
| *LEP* | rs2278815 | A/G | 43 | -5 | 18.61 | 0.80 |
| *LEP* | rs3828942 | G/A | 45 | -2 | 17.61 | 0.92 |
| *LEP* | rs7788818 | G/A | 6 | -9 | 25.24 | 0.73 |
| *LEPR* | rs10158579 | A/G | 13 | 7 | 16.51 | 0.67 |
| *LEPR* | rs1022981 | A/G | 25 | 15 | 13.44 | 0.27 |
| *LEPR* | rs1045895 | G/A | 40 | -5 | 11.73 | 0.65 |
| *LEPR* | rs10493380 | A/C | 19 | -30 | 14.71 | **0.04** |
| *LEPR* | rs11208659 | A/G | 10 | -6 | 19.30 | 0.78 |
| *LEPR* | rs1137100 | A/G | 24 | 16 | 13.49 | 0.23 |
| *LEPR* | rs1137101 | A/G | 46 | 7 | 27.62 | 0.79 |
| *LEPR* | rs11585329 | C/A | 15 | -3 | 16.01 | 0.83 |
| *LEPR* | rs1171267 | C/A | 34 | 12 | 15.41 | 0.43 |
| *LEPR* | rs1171278 | G/A | 18 | 13 | 18.22 | 0.47 |
| *LEPR* | rs1171279 | G/A | 27 | 0.4 | 12.96 | 0.98 |
| *LEPR* | rs12145690 | A/C | 45 | 7 | 12.36 | 0.55 |
| *LEPR* | rs12409877 | G/A | 39 | 13 | 11.81 | 0.26 |
| *LEPR* | rs1887285 | A/G | 9 | -21 | 19.33 | 0.28 |
| *LEPR* | rs1892534 | G/A | 38 | 2 | 11.77 | 0.85 |
| *LEPR* | rs1892535 | G/A | 18 | 18 | 15.21 | 0.23 |
| *LEPR* | rs2025805 | G/A | 47 | -15 | 11.62 | 0.18 |
| *LEPR* | rs3762274 | A/G | 39 | 12 | 12.49 | 0.33 |
| *LEPR* | rs3790426 | C/A | 24 | -5 | 13.53 | 0.71 |
| *LEPR* | rs3790433 | G/A | 26 | -17 | 12.99 | 0.19 |
| *LEPR* | rs3806318 | A/G | 28 | 7 | 13.57 | 0.60 |
| *LEPR* | rs4655537 | G/A | 36 | -3 | 17.66 | 0.87 |
| *LEPR* | rs4655802 | A/G | 41 | -5 | 11.80 | 0.69 |
| *LEPR* | rs6588147 | A/G | 32 | 21 | 12.51 | 0.09 |
| *LEPR* | rs6662904 | G/A | 48 | -13 | 11.62 | 0.25 |
| *LEPR* | rs6672331 | G/C | 3 | -11 | 40.58 | 0.79 |
| *LEPR* | rs6673324 | A/G | 49 | -11 | 13.60 | 0.42 |
| *LEPR* | rs6704167 | A/T | 45 | -8 | 13.48 | 0.54 |
| *LEPR* | rs7516341 | A/G | 37 | 8 | 11.86 | 0.49 |
| *LEPR* | rs8179183 | G/C | 18 | -16 | 14.73 | 0.29 |
| *LEPR* | rs9436297 | A/G | 14 | -5 | 20.95 | 0.81 |
| *LEPR* | rs9436301 | A/G | 24 | -5 | 13.42 | 0.70 |
| *LEPR* | rs9436740 | T/A | 28 | 18 | 13.08 | 0.18 |
| *LEPR* | rs9436746 | C/A | 40 | -2 | 14.54 | 0.91 |
| *LEPR* | rs970467 | G/A | 11 | 8 | 18.09 | 0.66 |
| *MC4R* | rs11872992 | G/A | 13 | -4 | 23.78 | 0.87 |
| *MC4R* | rs1943226 | A/C | 10 | -0.6 | 18.95 | 0.98 |
| *MC4R* | rs8093815 | G/A | 31 | 4 | 21.26 | 0.83 |
| *NMB* | rs1051168 | C/A | 29 | 14 | 12.65 | 0.27 |
| *NMB* | rs17598561 | G/A | 6 | 11 | 27.73 | 0.70 |
| *NMB* | rs2292462 | A/C | 47 | 7 | 11.66 | 0.53 |
| *NMB* | rs7180849 | G/A | 17 | -0.3 | 18.69 | 0.99 |
| *NPY* | rs12700524 | A/G | 14 | -15 | 16.58 | 0.38 |
| *NPY* | rs16135 | G/A | 7 | 23 | 26.86 | 0.39 |
| *NPY* | rs16141 | A/C | 49 | 12 | 18.28 | 0.50 |
| *NPY* | rs16148 | A/G | 34 | -0.2 | 14.16 | 0.99 |
| *NPY* | rs16472 | G/A | 9 | 9 | 20.87 | 0.68 |
| *NPY* | rs3025118 | C/A | 4 | -9 | 30.93 | 0.76 |
| *NPY* | rs5574 | G/A | 47 | 0.9 | 14.51 | 0.95 |
| *NPY* | rs9785023 | G/A | 50 | -6 | 13.76 | 0.68 |
| *NUCB2* | rs10741725 | C/A | 46 | -11 | 11.63 | 0.35 |
| *NUCB2* | rs10766383 | C/A | 28 | 14 | 21.73 | 0.51 |
| *NUCB2* | rs10832763 | A/G | 36 | 17 | 27.06 | 0.53 |
| *NUCB2* | rs12419530 | A/G | 4 | -17 | 37.53 | 0.65 |
| *NUCB2* | rs1330 | G/A | 33 | -16 | 17.40 | 0.37 |
| *NUCB2* | rs214075 | C/A | 41 | -8 | 11.80 | 0.52 |
| *NUCB2* | rs214082 | G/A | 41 | 12 | 11.98 | 0.33 |
| *NUCB2* | rs214086 | G/C | 42 | -10 | 12.71 | 0.43 |
| *NUCB2* | rs214105 | A/G | 28 | -20 | 13.14 | 0.14 |
| *NUCB2* | rs2634462 | G/A | 27 | -9 | 19.38 | 0.62 |
| *NUCB2* | rs7127347 | A/C | 13 | 7 | 16.64 | 0.67 |
| *NUCB2* | rs757081 | C/G | 32 | -13 | 13.26 | 0.33 |
| *POMC* | rs1866146 | A/G | 34 | -6 | 14.23 | 0.69 |
| *POMC* | rs3769671 | A/C | 3 | -30 | 41.92 | 0.48 |
| *POMC* | rs6545975 | A/G | 39 | -11 | 11.69 | 0.35 |
| *POMC* | rs6713532 | A/G | 23 | -12 | 13.63 | 0.38 |
| *POMC* | rs6719226 | G/C | 4 | -18 | 27.59 | 0.51 |
| *POMC* | rs6734859 | G/A | 13 | 17 | 16.62 | 0.32 |
| *POMC* | rs7565427 | G/A | 13 | 15 | 16.68 | 0.37 |
| *POMC* | rs7565877 | A/G | 11 | 17 | 18.66 | 0.36 |
| *POMC* | rs934778 | A/G | 30 | -5 | 12.59 | 0.72 |
| *PYY* | rs1058046 | G/C | 33 | -28 | 12.12 | **0.02** |
| *PYY* | rs1618809 | G/A | 37 | 5 | 14.59 | 0.73 |
| *PYY* | rs1662754 | A/T | 44 | -15 | 15.92 | 0.34 |
| *PYY* | rs1859223 | G/C | 16 | -38 | 23.59 | 0.10 |
| *PYY* | rs3744419 | G/A | 20 | 31 | 19.80 | 0.12 |
| *PYY* | rs8079623 | G/C | 11 | 31 | 18.75 | 0.10 |
| *PYY* | rs9907468 | G/A | 10 | -15 | 18.52 | 0.41 |

MAF: Minor Allele Frequency; SE: Standard Error

^1^ Values presented are the overall meta-analyzed regression coefficients and *P* values.
