# Supplementary material for: Genetic Polymorphisms in the Hypothalamic Pathway in Relation to Subsequent Weight Change – The DiOGenes Study
Source: PLoS One. 2011 Feb 24;6(2):e17436. doi: 10.1371/journal.pone.0017436 (PMC3044761; doi:10.1371/journal.pone.0017436)
Supplement: Table S6 — Interactions of 123 single nucleotide polymorphisms (SNPs) from the hypothalamic pathway with glycemic index (GI) and protein intake on weight gain (g/year) in random subcohort analysis (n = 6,566). (DOC) [file pone.0017436.s006.doc]

**Table S6. Interactions of 123 single nucleotide polymorphisms (SNPs) from the hypothalamic pathway with glycemic index (GI) and protein intake on weight gain (g/year) in random subcohort analysis (n = 6,566)^1^.**

| **Genes** | **SNPs** | **Major allele/ minor allele** | **MAF** | **1 minor allele × 1 unit GI** | | | **1 minor allele × 1 g protein** | | |
| --- | --- | --- | --- | --- | --- | --- | --- | --- | --- |
|  |  |  |  | **β** | **SE** | ***P* value** | **β** | **SE** | ***P* value** |
| *CCK* | rs10460960 | A/G | 11 | 3.58 | 5.54 | 0.52 | 0.21 | 7.94 | 0.98 |
| *CCK* | rs10865918 | A/C | 38 | -2.19 | 3.49 | 0.53 | -6.01 | 5.15 | 0.24 |
| *CCK* | rs11129949 | A/C | 12 | -6.19 | 5.33 | 0.25 | -4.21 | 7.73 | 0.59 |
| *CCK* | rs11571842 | G/A | 49 | 0.44 | 3.56 | 0.90 | -3.82 | 5.32 | 0.47 |
| *CCK* | rs747455 | G/A | 24 | 0.81 | 4.16 | 0.85 | 2.29 | 9.35 | 0.81 |
| *CCK* | rs7628795 | G/A | 41 | 2.82 | 3.57 | 0.43 | -3.96 | 5.19 | 0.45 |
| *CCK* | rs8192472 | G/A | 38 | 1.85 | 3.56 | 0.61 | 10.18 | 8.02 | 0.20 |
| *CCK* | rs9311317 | A/G | 25 | 4.27 | 4.08 | 0.30 | -8.18 | 9.25 | 0.38 |
| *CCKAR* | rs1573596 | G/A | 47 | 3.11 | 4.76 | 0.51 | -2.01 | 5.01 | 0.69 |
| *CCKAR* | rs2000978 | A/G | 17 | 5.20 | 6.61 | 0.43 | 2.06 | 6.35 | 0.75 |
| *CCKAR* | rs2854030 | G/A | 29 | -1.94 | 3.78 | 0.61 | -7.84 | 5.49 | 0.15 |
| *CCKAR* | rs7665027 | A/G | 15 | -2.45 | 4.68 | 0.60 | -1.83 | 6.81 | 0.79 |
| *CCKAR* | rs915889 | G/A | 7 | 9.54 | 9.05 | 0.29 | -4.27 | 19.03 | 0.82 |
| *mTOR* | rs1057079 | A/G | 26 | -3.24 | 4.96 | 0.51 | 7.04 | 6.98 | 0.31 |
| *mTOR* | rs1074078 | G/A | 33 | 0.63 | 3.68 | 0.86 | 3.99 | 5.48 | 0.47 |
| *mTOR* | rs12732063 | G/A | 5 | -6.35 | 12.89 | 0.62 | -12.90 | 14.59 | 0.38 |
| *mTOR* | rs1770345 | A/C | 47 | 14.23 | 10.36 | 0.17 | -3.13 | 11.39 | 0.78 |
| *GLP-1* | rs13416088 | G/A | 21 | 3.99 | 5.21 | 0.45 | -2.59 | 6.15 | 0.67 |
| *GLP-1* | rs3761656 | A/C | 8 | -7.67 | 6.34 | 0.23 | -1.93 | 9.40 | 0.84 |
| *GHRL* | rs10490815 | A/G | 29 | 0.18 | 3.79 | 0.96 | 2.83 | 5.44 | 0.60 |
| *GHRL* | rs11718213 | A/C | 10 | -2.38 | 9.92 | 0.81 | 8.60 | 8.31 | 0.30 |
| *GHRL* | rs1617161 | G/A | 11 | -0.23 | 8.95 | 0.98 | 2.07 | 7.691 | 0.79 |
| *GHRL* | rs1629816 | G/A | 38 | 7.50 | 6.29 | 0.23 | -0.087 | 6.93 | 0.99 |
| *GHRL* | rs17032621 | A/G | 14 | -3.03 | 4.84 | 0.53 | -8.54 | 7.27 | 0.24 |
| *GHRL* | rs171336 | C/A | 36 | 1.72 | 3.61 | 0.64 | -8.28 | 5.19 | 0.11 |
| *GHRL* | rs2619507 | A/G | 16 | -1.93 | 6.94 | 0.78 | 1.89 | 7.09 | 0.79 |
| *GHRL* | rs26802 | A/C | 33 | 0.25 | 6.61 | 0.97 | 7.58 | 5.32 | 0.15 |
| *GHRL* | rs27647 | A/G | 40 | 4.57 | 4.53 | 0.31 | -4.12 | 4.99 | 0.41 |
| *GHRL* | rs35683 | C/A | 48 | 7.68 | 3.46 | **0.03** | -6.07 | 4.95 | 0.22 |
| *GHRL* | rs35684 | A/G | 28 | -8.42 | 5.32 | 0.11 | 10.21 | 6.03 | 0.09 |
| *GHRL* | rs3755777 | G/C | 25 | -1.31 | 3.99 | 0.74 | -3.25 | 5.76 | 0.57 |
| *5-HT_1A_* | rs1423691 | A/G | 50 | -3.55 | 3.45 | 0.30 | -5.34 | 4.98 | 0.28 |
| *IL-6* | rs10242595 | G/A | 32 | -2.95 | 3.57 | 0.41 | 1.81 | 5.36 | 0.74 |
| *IL-6* | rs12700386 | G/C | 19 | -8.48 | 4.35 | 0.05 | 3.12 | 6.95 | 0.65 |
| *IL-6* | rs1800795 | C/G | 41 | 1.39 | 4.63 | 0.76 | -5.21 | 5.06 | 0.30 |
| *IL-6* | rs2069827 | C/A | 9 | 7.47 | 15.54 | 0.63 | 8.881 | 25.31 | 0.73 |
| *IL-6* | rs2069837 | A/G | 8 | -5.65 | 6.11 | 0.36 | 12.42 | 8.56 | 0.15 |
| *IL-6* | rs2069840 | G/C | 34 | -1.45 | 4.44 | 0.74 | 6.03 | 5.36 | 0.26 |
| *IL-6* | rs2069861 | G/A | 9 | -11.75 | 6.67 | 0.08 | 1.28 | 10.00 | 0.90 |
| *LEP* | rs11760956 | G/A | 37 | -5.75 | 5.15 | 0.26 | 1.89 | 6.96 | 0.79 |
| *LEP* | rs11763517 | A/G | 49 | -3.36 | 3.43 | 0.33 | 4.36 | 7.67 | 0.57 |
| *LEP* | rs2071045 | A/G | 24 | 0.66 | 4.02 | 0.87 | 11.07 | 5.87 | 0.06 |
| *LEP* | rs2278815 | A/G | 43 | -6.56 | 3.84 | 0.09 | -5.72 | 5.57 | 0.31 |
| *LEP* | rs3828942 | G/A | 45 | 2.49 | 11.39 | 0.83 | -5.26 | 8.14 | 0.52 |
| *LEP* | rs7788818 | G/A | 6 | -6.48 | 7.36 | 0.38 | -29.94 | 11.19 | **0.008** |
| *LEPR* | rs10158579 | A/G | 13 | 1.66 | 4.80 | 0.73 | -0.913 | 7.14 | 0.90 |
| *LEPR* | rs1022981 | A/G | 25 | 0.31 | 4.58 | 0.95 | -5.48 | 7.05 | 0.44 |
| *LEPR* | rs1045895 | G/A | 40 | -6.59 | 4.48 | 0.14 | -1.11 | 7.29 | 0.88 |
| *LEPR* | rs10493380 | A/C | 19 | -0.11 | 4.43 | 0.98 | 8.03 | 6.36 | 0.21 |
| *LEPR* | rs11208659 | A/G | 10 | 4.29 | 5.79 | 0.46 | -0.92 | 8.03 | 0.91 |
| *LEPR* | rs1137100 | A/G | 24 | 0.09 | 4.91 | 0.99 | -3.63 | 7.05 | 0.61 |
| *LEPR* | rs1137101 | A/G | 46 | -16.91 | 6.99 | **0.02** | -4.54 | 10.93 | 0.68 |
| *LEPR* | rs11585329 | C/A | 15 | 4.94 | 4.72 | 0.30 | 6.46 | 9.85 | 0.51 |
| *LEPR* | rs1171267 | C/A | 34 | 2.51 | 4.29 | 0.56 | -11.18 | 9.73 | 0.25 |
| *LEPR* | rs1171278 | G/A | 18 | 3.89 | 6.98 | 0.58 | 3.69 | 7.76 | 0.63 |
| *LEPR* | rs1171279 | G/A | 27 | 5.56 | 3.79 | 0.14 | 2.02 | 5.52 | 0.72 |
| *LEPR* | rs12145690 | A/C | 45 | 1.53 | 3.45 | 0.66 | 1.70 | 6.69 | 0.80 |
| *LEPR* | rs12409877 | G/A | 39 | 6.44 | 3.53 | 0.07 | -6.01 | 8.74 | 0.49 |
| *LEPR* | rs1887285 | A/G | 9 | -2.89 | 5.71 | 0.61 | -2.08 | 8.33 | 0.80 |
| *LEPR* | rs1892534 | G/A | 38 | -2.81 | 3.55 | 0.43 | -3.19 | 5.12 | 0.53 |
| *LEPR* | rs1892535 | G/A | 18 | -6.05 | 4.93 | 0.22 | -3.73 | 6.77 | 0.58 |
| *LEPR* | rs2025805 | G/A | 47 | -8.01 | 3.46 | **0.02** | 7.19 | 9.56 | 0.45 |
| *LEPR* | rs3762274 | A/G | 39 | -4.18 | 4.19 | 0.32 | -5.05 | 5.37 | 0.35 |
| *LEPR* | rs3790426 | C/A | 24 | 0.57 | 3.91 | 0.88 | -0.18 | 5.71 | 0.98 |
| *LEPR* | rs3790433 | G/A | 26 | -0.67 | 3.83 | 0.86 | 3.66 | 5.54 | 0.51 |
| *LEPR* | rs3806318 | A/G | 28 | 7.07 | 4.04 | 0.08 | -1.90 | 6.12 | 0.76 |
| *LEPR* | rs4655537 | G/A | 36 | -0.09 | 4.25 | 0.98 | 3.23 | 5.13 | 0.53 |
| *LEPR* | rs4655802 | A/G | 41 | -0.91 | 3.51 | 0.80 | -5.98 | 5.13 | 0.24 |
| *LEPR* | rs6588147 | A/G | 32 | 4.24 | 4.61 | 0.36 | -4.20 | 8.75 | 0.63 |
| *LEPR* | rs6662904 | G/A | 48 | -5.25 | 3.48 | 0.13 | 6.21 | 8.05 | 0.44 |
| *LEPR* | rs6672331 | G/C | 3 | -15.49 | 10.12 | 0.13 | 1.97 | 15.63 | 0.90 |
| *LEPR* | rs6673324 | A/G | 49 | -0.71 | 3.39 | 0.83 | 3.15 | 4.85 | 0.52 |
| *LEPR* | rs6704167 | A/T | 45 | -5.64 | 4.89 | 0.25 | 9.71 | 12.48 | 0.44 |
| *LEPR* | rs7516341 | A/G | 37 | -3.71 | 3.57 | 0.30 | -2.69 | 5.15 | 0.60 |
| *LEPR* | rs8179183 | G/C | 18 | 0.28 | 4.49 | 0.95 | -0.36 | 6.71 | 0.96 |
| *LEPR* | rs9436297 | A/G | 14 | -2.06 | 4.92 | 0.68 | 8.30 | 10.18 | 0.42 |
| *LEPR* | rs9436301 | A/G | 24 | 3.67 | 4.04 | 0.36 | 5.85 | 5.68 | 0.30 |
| *LEPR* | rs9436740 | T/A | 28 | 11.23 | 3.89 | **0.004** | -7.68 | 5.56 | 0.17 |
| *LEPR* | rs9436746 | C/A | 40 | 3.28 | 3.48 | 0.35 | -2.22 | 6.37 | 0.73 |
| *LEPR* | rs970467 | G/A | 11 | 5.85 | 6.99 | 0.40 | 2.12 | 10.42 | 0.84 |
| *MC4R* | rs11872992 | G/A | 13 | 0.11 | 5.00 | 0.98 | -2.26 | 12.47 | 0.86 |
| *MC4R* | rs1943226 | A/C | 10 | 7.12 | 5.55 | 0.20 | 4.51 | 8.20 | 0.58 |
| *MC4R* | rs8093815 | G/A | 31 | 3.78 | 3.69 | 0.31 | 5.55 | 5.96 | 0.35 |
| *NMB* | rs1051168 | C/A | 29 | -6.41 | 3.80 | 0.09 | 5.34 | 5.76 | 0.35 |
| *NMB* | rs17598561 | G/A | 6 | 2.15 | 11.39 | 0.85 | 11.79 | 11.70 | 0.31 |
| *NMB* | rs2292462 | A/C | 47 | 8.71 | 3.52 | **0.01** | -0.93 | 8.59 | 0.91 |
| *NMB* | rs7180849 | G/A | 17 | 24.92 | 4.81 | **2×10^-7^** | -19.57 | 8.52 | **0.02** |
| *NPY* | rs12700524 | A/G | 14 | 4.88 | 4.97 | 0.33 | 3.58 | 8.93 | 0.69 |
| *NPY* | rs16135 | G/A | 7 | 7.03 | 7.05 | 0.32 | -2.91 | 15.22 | 0.85 |
| *NPY* | rs16141 | A/C | 49 | -1.90 | 3.53 | 0.59 | -0.62 | 5.39 | 0.91 |
| *NPY* | rs16148 | A/G | 34 | 3.71 | 3.68 | 0.32 | 3.03 | 5.75 | 0.60 |
| *NPY* | rs16472 | G/A | 9 | -1.53 | 6.41 | 0.81 | -3.97 | 12.73 | 0.76 |
| *NPY* | rs3025118 | C/A | 4 | 2.64 | 11.94 | 0.83 | 0.56 | 25.16 | 0.98 |
| *NPY* | rs5574 | G/A | 47 | -5.05 | 3.43 | 0.14 | 0.97 | 6.36 | 0.88 |
| *NPY* | rs9785023 | G/A | 50 | 2.75 | 3.42 | 0.42 | -2.08 | 5.55 | 0.71 |
| *NUCB2* | rs10741725 | C/A | 46 | 5.99 | 3.43 | 0.08 | -5.85 | 5.07 | 0.25 |
| *NUCB2* | rs10766383 | C/A | 28 | -6.17 | 3.88 | 0.11 | 11.81 | 8.10 | 0.15 |
| *NUCB2* | rs10832763 | A/G | 36 | -1.23 | 4.23 | 0.77 | 11.95 | 7.97 | 0.13 |
| *NUCB2* | rs12419530 | A/G | 4 | 5.45 | 8.82 | 0.54 | -7.62 | 16.18 | 0.64 |
| *NUCB2* | rs1330 | G/A | 33 | 3.66 | 4.91 | 0.46 | -2.99 | 6.93 | 0.67 |
| *NUCB2* | rs214075 | C/A | 41 | 9.46 | 3.47 | **0.006** | -0.18 | 5.09 | 0.97 |
| *NUCB2* | rs214082 | G/A | 41 | -6.33 | 3.65 | 0.08 | 9.73 | 5.16 | 0.06 |
| *NUCB2* | rs214086 | G/C | 42 | 5.54 | 4.33 | 0.20 | -1.32 | 7.23 | 0.86 |
| *NUCB2* | rs214105 | A/G | 28 | 6.98 | 5.09 | 0.17 | 2.50 | 7.05 | 0.72 |
| *NUCB2* | rs2634462 | G/A | 27 | -0.35 | 3.85 | 0.93 | -7.76 | 8.28 | 0.35 |
| *NUCB2* | rs7127347 | A/C | 13 | 7.41 | 4.82 | 0.12 | -1.84 | 7.09 | 0.80 |
| *NUCB2* | rs757081 | C/G | 32 | 5.68 | 4.02 | 0.16 | 1.22 | 5.39 | 0.82 |
| *POMC* | rs1866146 | A/G | 34 | 9.56 | 5.98 | 0.11 | 0.27 | 5.61 | 0.96 |
| *POMC* | rs3769671 | A/C | 3 | -12.77 | 10.32 | 0.22 | -2.58 | 14.62 | 0.86 |
| *POMC* | rs6545975 | A/G | 39 | 1.99 | 3.46 | 0.57 | 9.41 | 5.16 | 0.07 |
| *POMC* | rs6713532 | A/G | 23 | 0.70 | 5.28 | 0.89 | -8.59 | 5.86 | 0.14 |
| *POMC* | rs6719226 | G/C | 4 | -20.52 | 11.49 | 0.07 | 1.40 | 12.25 | 0.91 |
| *POMC* | rs6734859 | G/A | 13 | 9.76 | 5.12 | 0.06 | 10.49 | 7.16 | 0.14 |
| *POMC* | rs7565427 | G/A | 13 | 12.16 | 5.87 | **0.04** | 11.46 | 7.23 | 0.11 |
| *POMC* | rs7565877 | A/G | 11 | -9.84 | 6.31 | 0.12 | 2.88 | 9.27 | 0.76 |
| *POMC* | rs934778 | A/G | 30 | -6.28 | 4.53 | 0.17 | 7.88 | 6.10 | 0.20 |
| *PYY* | rs1058046 | G/C | 33 | -2.27 | 7.06 | 0.75 | -4.77 | 6.83 | 0.49 |
| *PYY* | rs1618809 | G/A | 37 | 1.64 | 3.53 | 0.64 | -3.44 | 5.43 | 0.53 |
| *PYY* | rs1662754 | A/T | 44 | -6.08 | 6.16 | 0.32 | -8.34 | 4.97 | 0.09 |
| *PYY* | rs1859223 | G/C | 16 | -4.07 | 4.432 | 0.36 | 0.53 | 9.23 | 0.95 |
| *PYY* | rs3744419 | G/A | 20 | 4.54 | 4.48 | 0.31 | -6.89 | 6.66 | 0.30 |
| *PYY* | rs8079623 | G/C | 11 | -6.79 | 7.13 | 0.34 | -8.11 | 12.96 | 0.53 |
| *PYY* | rs9907468 | G/A | 10 | 0.02 | 5.33 | 0.99 | -2.95 | 15.97 | 0.85 |

MAF: Minor Allele Frequency; SE: Standard Error

^1^ Values presented are the overall meta-analyzed regression coefficients and *P* values.
